# Low-Velocity Impact Performance of Triax Glass Fabric Reinforced Poly(methyl methacrylate) Composites

**DOI:** 10.3390/ma19194065

**Published:** 2026-09-23

**Authors:** Yifan Wang, Xiaofeng Guo, Guangtao Li, Sen Wang, Jianmin Zhang, Simin Wang

**Affiliations:** 1 School of Intelligent Mechatronics Engineering, Zhongyuan University of Technology, Zhengzhou 450007, China; wiyifanwyf@163.com (Y.W.); lgtdqly@163.com (G.L.); 2 Longzihu New Energy Laboratory, Zhengzhou Institute of Emerging Industrial Technology, Zhengzhou 450000, China; wangsen_wy@163.com; 3 Beijing Key Laboratory of Ionic Liquids Clean Process, Institute of Process Engineering, Chinese Academy of Sciences, Beijing 100190, China; 4 Henan Winner Vibrating Equipment Co., Ltd., Xinxiang 453700, China; wangsimin@winnergroup.com.cn

**Keywords:** PMMA, triaxial glass fiber reinforced composite, finite element simulation, low-velocity impact

## Abstract

Impact resistance is vital for marine and protective structural composites. Conventional glass fiber/epoxy thermosets feature high specific strength but suffer poor recyclability. Herein, recyclable triaxial glass fiber-reinforced PMMA (Triax-GF/PMMA) laminates were manufactured, and quasi-static tensile, compressive and shear tests were conducted to obtain the fundamental mechanical constitutive parameters for the subsequent impact performance analysis. Low-velocity drop-weight impact (15–120 J) and compression-after-impact (CAI) experiments were carried out. Under a 50 J impact condition, the residual compressive strength of Triax-GF/PMMA reached 131.5 MPa, which was approximately 9.8% higher than that of the epoxy counterpart (119.8 MPa). Meanwhile, the peak impact load of Triax-GF/PMMA increased to 11.84 kN, compared with 9.98 kN for the epoxy system, indicating enhanced impact load-bearing capability. An ABAQUS/Explicit finite element model with Hashin failure criteria was validated by experimental results, with the relative errors of predicted peak loads maintained below 8%. Simulations at 30 J (non-penetration) and 120 J (penetration) showed milder deformation and slighter damage in Triax-GF/PMMA. A predictive model linking laminate thickness and perforation energy was further proposed. This work provides experimental and numerical support for the development of lightweight, recyclable, impact-resistant composites for potential marine applications.

## 1. Introduction

Fiber reinforced polymer (FRP) composites, characterized by their high specific strength, modulus, and excellent fatigue resistance, have been widely utilized in critical structural components such as hulls of high speed craft, deck overlays, cabin bulkheads, and offshore platforms [1,2,3]. This extensive application facilitates the lightweight structural design of ships and other marine engineering equipment. Currently, the structural components of these composites predominantly rely on thermosetting resin matrices, typical of which is epoxy resin. Upon crosslinking and curing, these resins form an irreversible three-dimensional network structure, rendering them exceedingly difficult to recycle or degrade at the end of their service life. Consequently, a vast majority of composite components from retired marine vessels and equipment are disposed of via landfilling or incineration. This practice not only causes a significant waste of material resources and environmental pollution but also contradicts the overarching goal of green and low carbon development in marine engineering [4,5,6]. Furthermore, throughout their entire service lifecycle, marine vessels are inevitably subjected to various impact loads, including dropped equipment on decks, collisions with floating debris along the hull, and impacts from floating ice during navigation in polar regions. Concealed damage induced by impacts at low velocity such as matrix cracking and interlaminar delamination can drastically compromise the residual structural strength, thereby posing a potential threat to navigational safety [7]. Therefore, developing fiber reinforced composites that simultaneously exhibit recyclability and superior impact resistance holds profound engineering value and practical significance.

In recent years, recyclable thermoplastic composites based on matrices such as PPS [8], PEEK [9], PA [10], and PMMA have emerged as a significant research hotspot. Comparative studies of thermoplastic and thermoset composites have further demonstrated that matrix type can significantly affect mechanical response, impact behavior, and damage tolerance [11]. Liu et al. [12] developed a PMMA/MMA binary liquid resin, achieving precise control over the viscosity of the resin system by adjusting the PMMA to MMA blending ratio. Building upon this, Gao et al. [13] demonstrated that PMMA, as a thermoplastic resin, can be efficiently recycled through melting or dissolution processes. Furthermore, De Tommaso et al. [14] pointed out that with the maturation of the recycled MMA (r-MMA) market, the closed-loop recycling of PMMA composites will exhibit excellent economic feasibility. Additionally, Ren et al. [15] experimentally demonstrated that thermoplastic PMMA sandwich composites can be recycled without the need for high-temperature pyrolysis or severe acid corrosion. These materials exhibit minimal mechanical degradation after multiple reshaping cycles, highlighting their prominent advantages in cyclic utilization.

In the structural design of marine applications, low-velocity impact performance is a critical metric for evaluating the operational safety of the structures [16,17]. Utilizing drop-weight impact tests combined with a Hashin progressive damage finite element model, Liu et al. [18] demonstrated that stitched-modified epoxy composites can suppress the propagation of impact-induced delamination, thereby improving the impact damage resistance of the epoxy system. Through impact testing coupled with finite element modeling, Gupta et al. [19] compared the impact damage evolution behaviors of epoxy laminates reinforced with different materials. They proved that under equivalent impact energies, basalt/epoxy composites exhibit a smaller damage area, although they are more susceptible to fiber breakage failure under high-energy impacts. By reviewing relevant experimental and simulation literature, Yang et al. [20] pointed out that thermoplastic matrix composites possess superior delamination resistance under impact compared to thermosetting epoxy composites. Fang et al. [21] investigated the cumulative damage evolution behavior of carbon fiber/epoxy laminates under repeated impacts. Through low-velocity impact tests integrated with two progressive damage finite element models in LS-Dyna, Kumar et al. [22] studied the mechanical responses and the evolution of resin-dominated delamination and matrix cracking in woven S2 glass fiber/epoxy composites under gradient low-velocity impacts ranging from 27.9 J to 109.7 J. Lastly, utilizing gradient drop-weight low-velocity impact tests combined with finite element modeling, Özsoy et al. [23] investigated the energy absorption characteristics and the entire damage evolution process in woven E-glass/epoxy laminates.

Impact loads exerted on marine structures, such as vessel decks and side shells, exhibit significant directional uncertainty. Consequently, traditional unidirectional or biaxial fabric-reinforced structures struggle to satisfy the load-bearing requirements of multi-directional impacts [24,25]. Triaxial glass fiber fabrics, characterized by their 0°/±45° multidirectional fiber architecture, can achieve in-plane load-bearing equilibrium across multiple directions. Furthermore, this intricate fiber network structure effectively suppresses the propagation of impact-induced damage, thereby demonstrating superior adaptability to the complex loading conditions of marine vessels.

A review of the literature indicates that current research on PMMA glass fiber-reinforced composites primarily focuses on the preparation of PMMA/MMA binary resins and their composites, general mechanical properties, as well as weather resistance and recyclability [26]. Research concerning the impact resistance of this material system remains relatively scarce; notably, there is a distinct gap in the systematic investigation of its low-velocity impact performance through both experimental testing and numerical simulation. In terms of mechanical characterization, previous studies [27] have evaluated the tensile, flexural, and short-beam shear strengths of PMMA glass fiber reinforced composites. However, the in-plane 0°/90°compressive and in-plane shear properties, which are indispensable constitutive parameters for the finite element simulation analysis of PMMA composites, have not yet been explored.

To this end, this study takes Triax-GF/PMMA laminates as the research subject to systematically investigate their low-velocity impact resistance from both experimental and numerical perspectives. Initially, the Triax-GF/PMMA laminates were fabricated using the VARI process. A complete set of fundamental mechanical parameters was obtained through 0°/90° tensile, compressive, and in-plane shear tests, providing the necessary constitutive model parameters for the subsequent impact finite element simulations. Subsequently, utilizing Triax-GF/Epoxy-manufactured with the exact same stacking sequence and molding process as a control group, gradient multi-energy low-velocity drop-weight impact and CAI tests were conducted. The dynamic impact responses, damage morphological characteristics, and differences in damage tolerance between the two systems were comparatively analyzed. Furthermore, an explicit dynamic finite element model incorporating the Hashin progressive failure criteria was established on the ABAQUS/Explicit platform. The reliability of this model was validated against the experimental load curves and damage morphologies. Based on the simulation results, the internal stress distribution and damage evolution mechanisms of the material under impact loading were thoroughly analyzed. Ultimately, a perforation energy predictive model for Triax-GF/PMMA laminates of varying thicknesses was developed.

This research is intended to provide robust experimental evidence and numerical references for the structural design of recyclable and impact-resistant composites in marine engineering. Although PMMA-based glass-fiber composites have attracted increasing attention because of their recyclability and promising mechanical performance [28,29], systematic understanding of their low-velocity impact response and corresponding damage-evolution mechanisms remains comparatively limited, particularly for triaxial glass-fiber architectures. Different from previous studies mainly focusing on the preparation, recyclability, and static mechanical properties of PMMA-based composites, this study provides a comprehensive investigation of Triax-GF/PMMA laminates by integrating multi-directional tensile, compressive, and shear characterization, low-velocity impact and compression-after-impact experiments, and experimentally validated progressive failure simulation. The findings provide new insights into the impact damage evolution mechanisms of recyclable composite laminates and offer guidance for the development of lightweight, impact-resistant composite structures for potential marine applications.

## 2. Material Preparation and Experimental Testing Methods

### 2.1. Experimental Materials

The methyl methacrylate (MMA) monomer (purity ≥ 99.9%, containing 30 ppm MEHQ inhibitor) utilized in this study was purchased from Shandong Keyuan Biochemical Co., Ltd., Jinan, China and was purified via vacuum distillation to remove the inhibitor prior to use. Benzoyl peroxide (BPO, 98% purity) and N, N-dimethylaniline (DMA, 99% purity) were supplied by Macklin and were used as received. Polymethyl methacrylate (PMMA) powder was acquired from Dongguan Hongxing New Material Co., Ltd., Dongguan, China The reinforcement material selected was a triaxial multi-axial stitched glass fiber fabric (EKT1215 series) provided by Chongqing International Composite Materials Co., Ltd., Chongqing, China. As illustrated in Figure 1, the fabric features a 0°/±45° triaxial architecture, wherein the 0° direction serves as the primary load-bearing layer with a fiber areal density of 709 g/m^2^, and the ±45° directions each possess an areal density of 250 g/m^2^. The three fiber orientations are not equally distributed; rather, the ±45° layers are symmetrically designed to mitigate layup coupling effects. The fiber type is E-glass with a monofilament diameter of 17 μm, and its surface was treated with a silane coupling agent to enhance the interfacial compatibility with the acrylate matrix. The epoxy resin system employed for the control group was TECHSTORM 180/185 (Hueber Advanced Materials Technology Co., Ltd., Shanghai, China), which was mixed according to the manufacturer’s recommended mass ratio of 100:20.

The PMMA powder and MMA monomer were mixed at a mass ratio of 24:100 in a sealed container. The mixture was then placed in a thermostatic shaker at 60 °C and continuously agitated for 12 h until the PMMA was completely dissolved, forming a homogeneous and transparent binary liquid resin. After cooling to room temperature, the reducing agent DMA and the oxidizing agent BPO were sequentially added under slow stirring at a molar ratio of MMA:BPO:DMA = 200:1.2:1 to ensure the complete dissolution of both agents, as depicted in Figure 2a (The selection of this ratio was based on previous research conducted by our group [30], which demonstrated its excellent overall performance).

For the preparation of the epoxy resin, the epoxy and curing agent were mixed in a beaker at a mass ratio of 100:20. The mixture was thoroughly stirred, as shown in Figure 2b, and subsequently placed in a vacuum chamber for 5 min to degas and eliminate air bubbles, thereby completing the preparation of both the PMMA and epoxy resin systems ready for infusion. Test results indicate that the viscosities of the resins used for the Triax-GF/PMMA and the Triax-GF/Epoxy during the VARI process are 105.37 mPa·s and 115.59 mPa·s, respectively.

### 2.2. Test Specimen Preparation

Both the Triax-GF/PMMA and the Triax-GF/Epoxy laminates were fabricated using the VARI process, as illustrated in Figure 3. During the preparation of the laminates, the materials were sequentially stacked in the following order: peel ply, triaxial glass fiber fabric, peel ply, and resin distribution mesh. Once the layup was completed, the spiral resin inlet tube and vacuum outlet line were positioned; the entire assembly was then covered with a vacuum bagging film and sealed with butyl sealant tape. The system was evacuated to a vacuum level of 0.095 MPa, after which the vacuum pump was turned off. This pressure was maintained for 30 min to verify airtightness; a vacuum drop of less than 0.5 kPa was deemed acceptable. The degassed resin was subsequently infused into the mold cavity at room temperature, with the vacuum level maintained at no less than 0.09 MPa throughout the infusion process. Upon completion of the infusion, both the resin inlet and vacuum outlet were sealed, and the mold was transferred to a forced-air drying oven for polymerization.

The temperature was maintained at a constant 35 °C for 12 h to ensure sufficient monomer conversion, followed by a post-curing stage at 80 °C for 4 h. Upon completion of the curing process, the mold was allowed to cool naturally to room temperature before demolding. The epoxy resin control group was infused utilizing the identical VARI process, with a curing profile of 50 °C for 6 h followed by 70 °C for 4 h.

Since the triaxial fabric composite consists of fibers oriented at 0° and ±45°, it exhibits favorable mechanical properties in both the X and Y directions. Accordingly, four layers of the triaxial fabric were laid up with the same ply orientation, and the impact specimens were fabricated via the vacuum infusion process.

The fiber volume fraction (V*_f_*) of the vacuum-infused Triax-GF/PMMA and Triax-GF/Epoxy laminates was calculated based on the measured fiber mass fraction and the densities of the constituent materials. The calculation was performed according to the following equation:

(1) $$V_{f}=\frac{w_{f}/\rho_{f}}{w_{f}/\rho_{f}+w_{m}/\rho_{m}}$$

where *w_f_* and *w_m_* represent the mass fractions of glass fiber and matrix, respectively, and *p_f_* and *p_m_* are the corresponding densities of glass fiber and matrix. The laminate mass was measured using an electronic balance, and the laminate thickness was measured using a digital micrometer at multiple locations. The average thickness was used for the calculation. The measured fiber volume fractions of the Triax-GF/PMMA and Triax-GF/Epoxy laminates were 85.76% and 84.87%, respectively. The laminate mass, thickness, and constituent densities are summarized in Table 1.

The elastic moduli and ultimate strengths of composites under 0° and 90° tension, compression, and in-plane shear are fundamental constitutive parameters required for constructing finite element models. However, existing research regarding the PMMA composites has predominantly focused on experimental tests focusing on 0° tension, flexure, and short-beam shear. These investigations were primarily conducted to evaluate the formulation process of the PMMA resin, its UV aging performance, and its recycling characteristics. Therefore, this study first investigates the relevant mechanical properties of the Triax-GF/PMMA.

## 3. Mechanics Performance Testing

### 3.1. Tensile Strength of the Samples

The mechanical properties of the specimens were evaluated using a TENSOR TEST-2 testing machine equipped with a 50 kN load cell (Sunstest Co., Ltd., Shenzhen, China). The 0° tensile specimens, with dimensions of 250 mm × 15 mm × 2 mm, were tested in accordance with the ISO 527-5 [31] standard at a loading rate of 2 mm/min. The 90° tensile specimens measured 250 mm × 25 mm × 4 mm and were tested according to the ISO 527-4 [32] standard at the same loading rate of 2 mm/min.

Figure 4a,b show photographs of the five Triax-GF/PMMA and Triax-GF/Epoxy specimens after the 0° tensile tests, respectively, while Figure 4c presents the tensile stress–strain curves. Similarly, Figure 4d,e show the specimens after the 90° tensile tests, and Figure 4f presents the tensile stress–strain curves. For the Triax-GF/PMMA composite, the tensile modulus and ultimate tensile strength in the 0° direction are 28.53 GPa and 587.10 MPa, respectively; in the 90° direction, the tensile modulus is 16.73 GPa, and the tensile strength is 144.11 MPa. Conversely, for the Triax-GF/Epoxy composite, the 0° tensile modulus and strength are 22.62 GPa and 474.68 MPa, respectively, whereas the 90° tensile modulus and strength are 10.81 GPa and 131.79 MPa, respectively.

### 3.2. Compression Tests of the Samples

In-plane compression tests of the composites were conducted in accordance with the ISO 14126 [33] standard. The specimen dimensions were 110 mm × 10 mm × 2 mm, and a standard matching anti-buckling support fixture was employed to prevent lateral buckling of the specimens during testing. The composites utilized a [0°/±45°] triaxial glass fiber fabric layup. When cutting the 0° longitudinal compression specimens, the 0° principal fiber direction of the fabric was aligned parallel to the loading axis. For the 90° transverse compression specimens, the laminate was rotated by 90° prior to cutting, orienting the 0° principal fibers perpendicular to the loading axis. Given the absence of 90° reinforcement yarns in this configuration, the compressive load is predominantly borne by the polymer matrix and the ±45° off-axis fibers. All compression tests were executed at a constant loading rate of 2 mm/min. Figure 5a,b show photographs of the five Triax-GF/PMMA and Triax-GF/Epoxy specimens after the 0° compression tests, respectively, while Figure 5c presents the t compression stress–strain curves. Similarly, Figure 5d,e show the specimens after the 90° compression tests, and Figure 5f presents the compression stress–strain curves. As derived from the curves, the Triax-GF/PMMA composite exhibits a 0° compressive modulus of 27.24 GPa and a compressive strength of 347.49 MPa; its 90° compressive modulus and strength are 13.02 GPa and 197.48 MPa, respectively. In comparison, the Triax-GF/Epoxy composite demonstrates a 0° compressive modulus of 19.00 GPa and a compressive strength of 317.79 MPa, while its 90° compressive modulus and strength are 9.45 GPa and 173.57 MPa, respectively.

### 3.3. In-Plane Shear Tests of the Samples

V-notched in-plane shear specimens were prepared using a universal cutting machine in accordance with the GB/T 28889 [34] standard. Figure 6a shows photographs of the five specimens from each material system after the in-plane shear tests, while Figure 6b presents the shear stress–strain curves. As depicted in Figure 6, the in-plane shear modulus of the Triax-GF/PMMA composite is 8.19 GPa, and its in-plane shear strength is 128.41 MPa. In comparison, the Triax-GF/Epoxy composite exhibits an in-plane shear modulus of 6.13 GPa and an in-plane shear strength of 119.44 MPa.

Synthesizing the experimental results from Figure 4, Figure 5 and Figure 6, the critical mechanical performance parameters for the Triax-GF/PMMA and the Triax-GF/Epoxy are summarized in Table 2. As the laminate lacks load-bearing fiber reinforcement in the through-thickness direction, the value of *G*_23_ is calculated based on the Chamis micromechanical model [35]. The in-plane longitudinal fibers exert a negligible effect on through-thickness shear properties, so *G*_13_ is equal to *G*_23_.

The test results indicate that, under the investigated experimental conditions and with the same triaxial glass fiber reinforcement and vacuum-assisted resin infusion molding process, the in-plane static mechanical properties of Triax-GF/PMMA composites are better than those of Triax-GF/Epoxy composites. In the 0°principal load-bearing direction, Triax-GF/PMMA exhibits higher tensile and compressive module than the epoxy-based composite system, with increases of 26.1% and 43.4%, respectively. Meanwhile, its tensile and compressive strengths are improved by 23.7% and 9.3%, which indicates an enhanced load-bearing capability of the PMMA-based laminates and suggests effective stress transfer between the PMMA matrix and glass fibers under static loading conditions.

In the 90°transverse direction and in-plane shear state, Triax-GF/PMMA still maintains a significant performance advantage. Specifically, its transverse tensile modulus, transverse compressive modulus, and in-plane shear modulus are 54.8%, 37.8%, and 33.6% higher than those of Triax-GF/Epoxy, respectively, with corresponding strength indicators also improved to varying degrees. The in-plane shear properties obtained in this study reflect the shear load-bearing capability of the laminates and provide essential constitutive parameters for the subsequent progressive failure simulation. Although the improved shear performance suggests favorable mechanical interaction between the PMMA matrix and glass fibers, the intrinsic interfacial fracture behavior and long-term interfacial durability require further investigation.

It should be noted that the higher quasi-static mechanical properties of Triax-GF/PMMA do not, by themselves, establish superior impact damage tolerance. Low-velocity impact behavior is governed by multiple interacting factors, including matrix toughness and ductility, fiber–matrix adhesion, interlaminar fracture resistance, and the initiation and propagation of different damage modes. Therefore, the quasi-static mechanical properties measured in this study are primarily regarded as baseline material characteristics and constitutive inputs for the subsequent numerical analysis.

## 4. Low-Velocity Drop-Weight Impact Tests and Results

### 4.1. Low-Velocity Drop-Weight Impact Tests

The low-velocity impact tests were conducted in accordance with the ASTM D7136 [36] standard. The fabricated laminates were cut into standard rectangular specimens with dimensions of 150 mm × 100 mm × 4 mm using a universal cutting machine.

The low-velocity impact tests were performed on a SANS drop-weight impact testing machine. As illustrated in Figure 7, this equipment primarily consists of five components: the main impact frame, the drop-weight impactor assembly, the pneumatic specimen clamping fixture, the control panel, and the data acquisition and processing computer. Figure 7a presents a magnified detail of the fixture structure. The upper section features the impactor, which can be configured with varying impact masses to attain predetermined impact energies via free fall. The lower section is equipped with a pneumatic specimen clamping fixture that employs pneumatic cylinders to securely clamp all four edges of the laminate specimen. This mechanism prevents any slippage of the specimen during the impact event, thereby satisfying the rigorous boundary constraint requirements for low-velocity impact testing. Figure 7b displays the complete system. The main testing apparatus is housed within an enclosed protective cabinet to prevent the ejection of debris, ensuring experimental safety. The exterior of the cabinet is fitted with a touch-screen control panel utilized to configure test parameters, including drop height and impact mass. The accompanying computer terminal on the right is dedicated to the real-time acquisition of transient data such as force, displacement, time, and energy throughout the impact process.

To investigate the material response across a comprehensive energy spectrum, four impact energy gradients were established: 15 J, 30 J, 50 J, and 120 J. A hemispherical steel impactor with a diameter of 16 mm was utilized, and the total impactor mass was initially set to 5.5 kg. However, due to the operational limits of the testing apparatus, the impactor mass was increased to 13.5 kg specifically for the 120 J impact tests. The various initial impact kinetic energies were achieved by systematically adjusting the drop height. For all mechanical tests conducted in this study, five valid specimens were evaluated under each identical testing condition to ensure the statistical reliability of the data.

### 4.2. Low-Velocity Drop-Weight Impact Test Results

Figure 8, Figure 9 and Figure 10 present the dynamic impact response curves of the Triax-GF/PMMA and the Triax-GF/Epoxy under the four impact energy levels of 15 J, 30 J, 50 J, and 120 J.

As illustrated in Figure 8a,b, the peak loads of the Triax-GF/PMMA under impact energies of 15 J, 30 J, 50 J, and 120 J are 6.01 kN, 8.56 kN, 11.84 kN, and 19.35 kN, respectively. For the Triax-GF/Epoxy, the peak loads under the identical energy levels are 5.80 kN, 8.16 kN, 9.98 kN, and 15.29 kN, respectively.

For both the PMMA-based and epoxy-based composites, the peak impact load consistently increases with the elevation of impact energy (15 J, 30 J, 50 J, and 120 J). All force-time curves demonstrate typical impact response characteristics, initially ascending to a peak load before gradually decaying. Under the low-to-medium energy conditions of 15–50 J, the curve profiles remain relatively smooth and stable. In contrast, under the 120 J high-energy impact, both sets of curves exhibit pronounced load oscillations, indicating that high-energy loading induces internal micro-damage and stress fluctuations within the materials. Furthermore, the overall impact duration for the PMMA system is concentrated within 0–7 ms, whereas the epoxy system experiences a more prolonged impact duration (0–11 ms), signifying a more gradual load attenuation in the epoxy matrix post-impact.

According to the force-time curves across various energy gradients presented in Figure 8, under all impact conditions ranging from 15 J to 120 J, the PMMA-based composites consistently exhibit higher peak impact loads compared to the epoxy-based system at identical energy levels, demonstrating a superior instantaneous impact load-bearing capacity. Furthermore, the PMMA system requires less time to reach the peak load, indicating a more rapid impact response rate and a quicker adaptation to the applied impact loads. Additionally, the overall impact duration for the PMMA system is shorter, enabling it to rapidly complete the dynamic mechanical response and the subsequent unloading process. Although load oscillations are observed under high-energy impacts, the PMMA composite maintains a prominent advantage in peak load-bearing capability, ultimately revealing that its dynamic impact resistance is superior to that of the epoxy-based composite.

As illustrated in Figure 9a,b, the ascending phase of the energy-time curves corresponds to the conversion of the impactor’s kinetic energy into the elastic deformation energy and damage dissipation energy of the laminates. Conversely, the descending phase corresponds to the release of elastic deformation energy during the rebound of the impactor. The stable plateau at the end of the curve represents the irreversible damage dissipation energy of the laminates. Under the 15 J, 30 J, and 50 J conditions, the dissipated energy of both materials increases monotonically with the elevation of impact energy. The curves initially ascend rapidly to a peak before slowly descending and ultimately stabilizing. The final dissipated energies of the Triax-GF/PMMA composite under the aforementioned conditions are 2.51 J, 10.71 J, and 23.86 J, respectively, while those of the Triax-GF/Epoxy system are 2.47 J, 9.68 J, and 26.31 J, respectively. Under the 15 J and 30 J impacts, the damage dissipation energy of Triax-GF/PMMA is slightly higher than that of Triax-GF/Epoxy. However, as the energy increases to 50 J, the absorbed energy of Triax-GF/Epoxy surpasses that of Triax-GF/PMMA. This is attributed to the applied load exceeding the critical damage threshold of the Triax-GF/Epoxy system, triggering extensive interlaminar delamination and massive fiber breakage, which constitute irreversible damage. Under the 120 J impact, the energy-time curves of both systems no longer exhibit a descending rebound phase; instead, they continuously ascend and ultimately converge at 120 J. This indicates that the initial kinetic energy of the impactor has been entirely absorbed by the laminates and converted into the fracture energy of penetration and frictional dissipation energy, demonstrating that the specimens have experienced complete perforation failure under this condition.

As observed from the energy-time curves in Figure 9, under identical impact energy gradients, the PMMA-based composites exhibit a faster energy absorption response rate and reach the peak energy absorption earlier, indicating a higher initial impact energy conversion efficiency. Under low-to-medium energy conditions, the PMMA system achieves a higher peak absorbed energy and demonstrates better energy retention performance during the energy descending phase. Under the 120 J high-energy impact, it can rapidly complete the full absorption of the impact energy and maintain a stable, saturated state. The overall energy absorption efficiency and dynamic energy response characteristics of the PMMA-based composites are superior to those of the epoxy-based system. This ultimately suggests a relatively favorable impact resistance of the PMMA material, particularly in its capacity for the rapid dissipation of impact energy.

Figure 10 displays the impact force-displacement response curves for the Triax-GF/PMMA and the Triax-GF/Epoxy under the four impact energy levels. Herein, the area enclosed by the curve and the horizontal axis corresponds to the total energy absorbed during the impact event. The slope of the ascending loading phase reflects the dynamic bending stiffness of the laminates, whereas the characteristics of the unloading phase and the final residual displacement indicate the extent of cumulative damage and irreversible deformation.

Under the three non-penetrating impact conditions of 15 J, 30 J, and 50 J, the force-displacement curves of both materials exhibit a closed hysteresis loop, comprising an approximately linear ascending loading phase and a nonlinear descending unloading phase. The final residual displacement gradually increases with the elevation of impact energy, which corresponds to the progressive accumulation of internal damage within the laminates. Comparing the characteristic parameters, the peak displacements of the Triax-GF/PMMA composite under 15 J, 30 J, and 50 J are approximately 6.39 mm, 8.35 mm, and 9.91 mm, respectively; in contrast, the peak displacements of the Triax-GF/Epoxy composite under the identical energies are approximately 6.80 mm, 8.11 mm, and 9.77 mm, respectively. Under the 15 J low-energy impact, the Triax-GF/Epoxy system demonstrates a larger peak displacement but a lower peak load, indicating that its dynamic bending stiffness is inferior to that of the Triax-GF/PMMA system. As the impact energy increases to 30 J and 50 J, the peak displacements of the two materials gradually converge; however, the peak load of Triax-GF/PMMA remains consistently higher than that of Triax-GF/Epoxy. This indicates that under a similar degree of bending deformation, the PMMA matrix can more efficiently transfer the applied load to the triaxial fiber reinforcement phase, thereby fully exploiting the load-bearing potential of the fibers. Consequently, the PMMA-based laminates exhibit superior specific stiffness and impact load-bearing capabilities.

When the impact energy is elevated to 120 J, both materials undergo complete perforation failure, and their force-displacement curves no longer form closed hysteresis loops. Following the peak load, the force declines rapidly concurrently with massive fiber breakage and catastrophic structural failure. The final displacement at the end of the curve directly corresponds to the complete penetration depth of the impactor. The Triax-GF/PMMA composite exhibits a peak displacement of approximately 13.56 mm, and its load profile in the peak region displays pronounced serrated oscillation characteristics. In contrast, the Triax-GF/Epoxy composite demonstrates a peak displacement of approximately 14.3 mm, exhibiting a less severe load oscillation amplitude; however, its post-peak load attenuation is significantly more rapid, ultimately resulting in a larger overall penetration displacement.

According to the force-displacement curves across various impact energies presented in Figure 10, under all energy gradients ranging from 15 J to 120 J, the PMMA-based composites can withstand higher impact loads at a given displacement. Furthermore, their peak impact loads consistently surpass those of the epoxy-based system. The PMMA composite reaches its ultimate load-bearing peak at a smaller displacement, indicating greater structural stiffness and enhanced deformation resistance. This capability allows the material to achieve superior impact load-bearing performance with minimal structural deformation, making it less susceptible to the formation of extensive impact indentations. Comparing the morphological characteristics of the curves, the ascending loading phase of the PMMA composite exhibits a steeper slope, highlighting its prominent dynamic rigidity. Possessing a relatively distinct advantage in peak load-bearing capacity, the PMMA system demonstrates favorable performance in both impact load-bearing and deformation control, ultimately reflecting a somewhat superior dynamic impact resistance compared to the epoxy-based composite.

### 4.3. Compression After Impact Residual Strength Analysis

To further quantify the degradation of the structural residual load-bearing capacity induced by low-velocity impact damage and to comprehensively evaluate the damage tolerance of the composites, CAI tests were conducted on the Triax-GF/PMMA and Triax-GF/Epoxy laminates subjected to impact energies of 15 J, 30 J, 50 J, and 120 J. The CAI tests were executed in accordance with the ASTM D7137 [37] standard. The post-impact specimens were mounted into a specialized anti-buckling CAI test fixture. This fixture provides simply supported boundary conditions along the four edges of the specimen, thereby preventing macroscopic global buckling of the laminates during the in-plane compressive loading process. The tests were performed utilizing a universal testing machine at a constant loading rate stipulated by the standard, continuing until complete compressive failure of the specimens occurred. The maximum failure load was recorded to calculate the ultimate residual compressive strength.

The experimental results for the ultimate residual compressive strength are presented in Figure 11. As the initial impact energy increases from 15 J to 120 J, the ultimate residual compressive strength of both material systems exhibits a non-linear degradation trend. Under the 15 J low-energy impact, the internal damage to the materials is relatively minimal, and the residual strengths of Triax-GF/PMMA and Triax-GF/Epoxy are maintained at a high level of 286.1 MPa and 281.5 MPa, respectively. When the impact energy is elevated to 30 J and 50 J, the progressive internal damage accumulation intensifies, resulting in a significant divergence in the residual strengths of the two materials. Particularly under the 50 J condition, the Triax-GF/PMMA composite retains a compressive strength of 131.5 MPa, whereas the Triax-GF/Epoxy sharply deteriorates to 119.8 MPa. Under the 120 J high-energy complete perforation condition, the macroscopic structures of both composites suffer severe catastrophic damage; nevertheless, the residual strength of Triax-GF/PMMA (46.4 MPa) remains superior to that of Triax-GF/Epoxy (44.8 MPa).

Across all tested energy gradients, the post-impact residual strength of the PMMA-based composites consistently surpasses that of the epoxy-based control group. Particularly under the 50 J impact energy condition, the residual compressive strength of Triax-GF/PMMA is approximately 9.8% higher than that of Triax-GF/Epoxy. Even under the extreme 120 J perforation condition, the PMMA system maintains higher residual load-bearing capability than the epoxy counterpart, indicating improved post-impact structural performance under the investigated conditions. After being subjected to equivalent impact kinetic energies, the Triax-GF/PMMA laminates are capable of retaining a larger proportion of their effective load-bearing cross-sectional area and global bending stiffness. Macroscopically, this translates into a higher ultimate residual compressive strength and enhanced structural reliability during service. However, the long-term structural reliability of PMMA-based composites in marine environments requires further validation considering possible environmental degradation effects on matrix properties and fiber–matrix interfaces.

## 5. Finite Element Simulation Analysis

To simulate the dynamic response and progressive failure process of the composite materials subjected to low-velocity drop-weight impact, and to analyze the internal stress distribution and damage evolution mechanisms under impact loading, a finite element analysis (FEA) model was established in this study utilizing the ABAQUS/Explicit solver.

### 5.1. Finite Element Model

The geometric dimensions of the model were set to 150 mm × 100 mm, consistent with the actual experimental specimens. For the composite laminate, the stacking sequence was defined as [0°/45°/−45°]s. The drop-weight impactor was simplified as a hemispherical rigid body with a diameter of 16 mm. The initial clearance between its outer surface and the upper surface of the specimen was established at 0.1 mm to simulate the incipient contact state upon release during the test. Only the translational degree of freedom along the impact direction was retained, as depicted in Figure 12d. A mass of 5.5 kg was assigned to the impactor via a concentrated mass element at the rigid body’s reference point. Initial velocities of 2.3355 m/s, 3.3029 m/s, and 4.2640 m/s were applied to the impactor along the impact axis, equivalent to the prescribed impact energies of 15 J, 30 J, and 50 J, respectively. To maintain consistency with the experimental configuration, the impactor mass was adjusted to 13.5 kg when simulating the 120 J penetration energy.

To balance computational accuracy and solution efficiency, a partitioned meshing strategy was adopted for the laminate, as illustrated in Figure 12b. Specifically, a local high-density mesh was applied to the central impact region, and the laminate was discretized with three elements per physical ply along the thickness direction. Eight-node quadrilateral in-plane general-purpose continuum shell elements (SC8R) were employed for the model. This element type utilizes a reduced integration scheme and incorporates a stiffness-based hourglass control algorithm, thereby effectively suppressing the occurrence of zero-energy modes.

The boundary conditions of the simulation were established in accordance with the actual drop-weight impact tests, as depicted in Figure 12c. The supporting base was defined as a rigid body and coupled to a reference point (RP) via a rigid body constraint. A fully fixed boundary condition (U1 = U2 = U3 = UR1 = UR2 = UR3 = 0) was applied to this RP, constraining all translational and rotational degrees of freedom. The mechanical interaction between the impactor and the specimen was modeled utilizing the General Contact algorithm. The normal contact behavior was defined as “Hard” contact, which allows for subsequent separation of the interfaces following initial contact. The tangential behavior was governed by a penalty friction formulation, with the friction coefficient specified as 0.2. Furthermore, surface-based cohesive behavior was assigned to the interfaces between adjacent composite plies to account for interlaminar damage [38,39]. The Hashin failure criteria were used exclusively to describe intralaminar damage initiation and evolution within the composite plies, including fiber tensile failure, fiber compressive failure, matrix tensile failure, and matrix compressive failure. Thus, in the present model, the two complementary damage formulations were employed to distinguish intralaminar and interlaminar failure mechanisms.

The interface response was described by a traction–separation relationship, in which the interfacial strengths *t_n_*^0^ and *t_s_*^0^ govern the initiation of normal and shear-dominated interface damage, respectively, whereas the critical fracture energies *G_IC_* and *G_IIC_* control the subsequent propagation of Mode-I and Mode-II interfacial damage. The cohesive-interface parameters adopted for the two composite systems are summarized in Table 3.

### 5.2. Failure Criteria of the Samples

To accurately capture the in-plane damage evolution behavior of the composite laminates under complex stress states, the two-dimensional (2D) Hashin progressive failure criterion was incorporated in this study. This criterion comprehensively accounts for the interactive effects of multi-axial stresses and effectively predicts the degradation effects of four fundamental in-plane failure modes—namely, fiber tension, fiber compression, matrix tension, and matrix compression—on the macroscopic mechanical response of the material. The specific damage initiation criteria are defined as follows [40]:

Fiber tensile failure(*σ*_11_ ≥ 0):

(2) $${\left(\frac{\sigma_{11}}{X_{T}}\right)}^{2}+{\left(\frac{\sigma_{12}}{S_{12}^{2}}\right)}^{2}\geq 1$$

Fiber compressive failure(*σ*_11_ < 0):

(3) $${\left(\frac{\sigma_{11}}{X_{C}}\right)}^{2}\geq 1$$

Matrix tensile failure(*σ*_22_ + *σ*_33_ ≥ 0):

(4) $${\left(\frac{\sigma_{22}}{Y_{T}}\right)}^{2}+{\left(\frac{\sigma_{12}}{S_{12}^{2}}\right)}^{2}\geq 1$$

Matrix compressive failure(*σ*_22_ + *σ*_33_ < 0):

(5) $${\left(\frac{\sigma_{22}}{2S_{23}}\right)}^{2}+\left[{\left(\frac{Y_{C}}{2S_{23}}\right)}^{2}-1\right]\frac{\sigma_{22}}{Y_{C}}+{\left(\frac{\sigma_{12}}{S_{12}}\right)}^{2}\geq 1$$

*σ*_11_, *σ*_22_, and *σ*_12_ denote the effective normal stresses along the principal material axes and the effective in-plane shear stress, which are introduced to account for the reduction in effective load-bearing area induced by microcrack propagation. *X_T_* and *X_C_* correspond to the longitudinal tensile and compressive strengths, respectively; *Y_T_* and *Y_C_* represent the transverse tensile and compressive strengths, *S*_12_ and *S*_23_, respectively; and are the longitudinal and transverse shear strengths. Once the damage index F of any failure mode reaches unity, damage initiation is identified at the corresponding integration point, which subsequently activates the subsequent stiffness degradation evolution routine.

During the modeling procedure, the built-in Hashin damage model in Abaqus/Explicit is adopted to invoke the constitutive model for composite materials, whereby appropriate material properties are assigned to composite shells. The detailed material parameters are summarized in Table 1 above.

### 5.3. Model Validation

To verify the predictive capability of the established model for Triax-GF/PMMA and Triax-GF/Epoxy composites under various impact energies, the experimental and simulated peak forces of the two materials at distinct energy levels are compared in Table 4. The relative errors of peak impact force remain below 8% across all applied impact energies, which validates the reliability of the numerical simulation framework

To further verify the accuracy and reliability of the constructed explicit dynamic finite element model, a systematic comparative analysis between numerical predictions and experimental measurements is conducted from two perspectives, namely macroscopic dynamic mechanical responses and local damage morphologies, taking the impact loading condition of 30 J as a representative case.

As illustrated in Figure 13, the load curves obtained from numerical simulation generally agree with the experimental measurements in terms of overall evolutionary tendency. The experimentally measured peak loads of Triax-GF/PMMA and Triax-GF/Epoxy composites are 8.56 kN and 8.16 kN, while the numerically predicted peak loads are 8.38 kN and 8.09 kN, respectively. The relative deviations of peak force for both materials fall within an acceptable engineering margin, demonstrating that the developed damage model can precisely characterize the stiffness evolution behavior of Triax-GF/PMMA and Triax-GF/Epoxy composites subjected to impact loading.

Following the global load verification, Figure 14 presents a comparative analysis of failure morphologies for Triax-GF/PMMA composite specimens. On the impact face, localized indentations and matrix crushing zones induced by extreme extrusion of the impactor are observed in experiments. The contour plots of fiber compressive damage output from simulations accurately reproduce such phenomena, and the damaged regions are well consistent with the indentation morphologies captured experimentally. In terms of the rear face, the failure features of experimental specimens match those predicted by the finite element model. Such results confirm that the explicit dynamic computational framework adopted in this work can reconstruct the entire complex dynamic failure process of the composite under impact loading with high fidelity, laying a solid and credible foundation for the subsequent investigation of energy dissipation mechanisms.

### 5.4. Damage Mechanisms of Composite Materials

To investigate the damage evolution laws of composite materials under impact loading, impact tests at energy levels of 30 J and 120 J are selected as research cases in this study. The internal stress distribution, damage, and deformation characteristics inside the composites subjected to impact loads are analyzed by combining morphological observations and numerical-experimental comparisons.

Figure 15 shows the simulated field distributions and experimental impact morphologies of Triax-GF/PMMA and Triax-GF/Epoxy composites under 30 J low-velocity impact loading. Specifically, Figure 15a,d present the simulated contours of the impact-face stress, back-face stress, overall through-thickness section stress, and out-of-plane displacement of the first-layer panel for the two materials, respectively. Figure 15b,c,e,f correspond to the impact-face and back-face morphologies of the two types of specimens after impact, respectively.

In terms of stress characteristics, both systems conform to the bending stress distribution of laminated plates, with the maximum tensile stress located at the center of the back face and compressive stress concentrated in the contact region of the impactor. The peak Mises stress of Triax-GF/PMMA is approximately 1205 MPa, and the high-stress region exhibits a dumbbell-shaped localized distribution along the 0° fibers, reflecting the efficient load transfer at the PMMA matrix–fiber interface. The peak Mises stress of Triax-GF/Epoxy is approximately 1102 MPa, and its stress distribution is more diffuse. This is because the brittle epoxy interface tends to initiate microcracks prematurely, which dissipates part of the impact energy while reducing the degree of local stress concentration. As shown in Figure 15c,f, localized impact damage occurred on the back face of the specimens, which is consistent with the simulation results presented in Figure 15a,d. In terms of deformation characteristics, the out-of-plane displacements of both materials exhibit a centrally symmetric bowl-shaped distribution, with the maximum deflection located at the center of the back face. The maximum out-of-plane displacement of Triax-GF/PMMA is approximately 8.8 mm, while that of Triax-GF/Epoxy is approximately 8.4 mm; the difference in deflection is consistent with the variation in their flexural stiffness.

Under 30 J low-velocity impact, Triax-GF/PMMA exhibits a higher peak Mises stress and a more localized high-stress region, indicating that its fiber–matrix interface load transfer efficiency is superior to that of Triax-GF/Epoxy. The difference in deflection between the two materials conforms to the flexural stiffness behavior. Meanwhile, Triax-GF/PMMA shows a more confined damage region after impact, with crack propagation effectively suppressed, resulting in better overall impact-resistant load-bearing performance.

Figure 16 compares the simulated field distributions and experimental failure morphologies of Triax-GF/PMMA and Triax-GF/Epoxy composites under 120 J high-energy penetration impact. For both materials, the extreme stress values are concentrated in the fiber fracture regions corresponding to the penetration cracks. The peak Mises stress of Triax-GF/PMMA reaches 1371 MPa, higher than the 1265 MPa of Triax-GF/Epoxy. The high stress is directionally distributed along the fiber orientation, reflecting the firm bonding of the PMMA matrix–fiber interface and the stronger load transfer capability. In the epoxy system, the stress distribution is more diffuse, and the interface is prone to initiate damage over a large area, which disperses the stress and makes it difficult to achieve efficient stress transfer and load bearing.

In terms of deformation characteristics, the maximum out-of-plane deflection of both materials occurs on the back face at the impact center. The maximum central deflection of Triax-GF/PMMA is 14.2 mm, which is smaller than the 14.9 mm of the epoxy system. The larger deformation of the epoxy system is attributed to extensive interlaminar delamination induced by the impact, which significantly degrades the structural flexural stiffness. In contrast, the PMMA system effectively suppresses the propagation of interlaminar delamination, resulting in superior stiffness retention.

The physical damage morphology further illustrates this difference: the impact face of the PMMA specimen exhibits only localized concentrated impact damage, and the crack propagation on the back face is limited in extent; in contrast, the epoxy specimen shows extensive delamination damage over a large area on the impact face and large-scale fiber tearing on the back face. Under high-energy impact loading, the Triax-GF/PMMA composite exhibits relatively stronger resistance to delamination and crack propagation, better preservation of overall structural integrity, and certain advantages in comprehensive high-energy impact protection performance.

### 5.5. Analysis of Perforation Energy of Composite Laminates with Different Thicknesses

To further investigate the influence of laminate thickness on the impact resistance and perforation threshold of the composites, this section numerically predicts the drop-weight impact process of Triax-GF/PMMA composite laminates with different thicknesses based on finite element simulation data. Figure 17a presents the relationship between absorbed energy and impact energy for laminates with thicknesses of 4 mm, 6 mm, and 8 mm under various initial impact energies, with the experimental data of 4 mm laminates included for model validation. The solid diagonal line in the figure denotes the complete absorption line, which corresponds to the state of full energy dissipation and complete perforation. As shown in Figure 17a, under low- and medium-energy impact conditions of 15 J, 30 J, and 50 J, all three sets of laminates remain free of penetration failure, with all data points located below the complete absorption line. At an identical input energy level, the irreversible absorbed energy of the laminates decreases gradually as the laminate thickness increases. This phenomenon is primarily attributed to the enhanced flexural stiffness brought by increased thickness, which restricts the deflection deformation during impact. Accordingly, energy dissipation mechanisms including matrix plastic deformation, fiber fracture, and interlaminar delamination are suppressed. A larger proportion of the initial kinetic energy is converted into elastic strain energy stored in the structure, which is subsequently released during the unloading stage and drives the rebound of the impactor. When the input energy rises to 120 J, the data point of the 4 mm-thick laminate nearly falls on the complete absorption line, with a simulated absorbed energy of 119 J. This indicates that the impact kinetic energy is almost fully dissipated by the material, resulting in complete perforation failure of the structure, which agrees well with the experimental results. By contrast, the 6 mm and 8 mm laminates exhibit absorbed energy values of 107.8 J and 97.3 J, respectively, both of which remain below the complete absorption line. This result verifies that the thicker laminates have not been perforated under this energy level and still maintain a certain impact resistance margin.

Based on the above simulation results, a prediction model for the perforation energy of the composite as a function of target plate thickness was further established, as shown in Figure 17b. In the figure, the red scatter points represent the simulated perforation energy values, and the black solid line is the prediction curve output by the simulation model, expressed as y = 2.6214x^2^ + 17.9329x + 8.84. The results indicate that the prediction model achieves a high degree of fit with the simulation data, with a correlation coefficient R^2^ of 0.997, suggesting that the finite element numerical model possesses satisfactory accuracy and reliability in predicting the ultimate damage tolerance of the material. From the perspective of the evolution trend, the perforation energy exhibits a nonlinear increasing tendency with increasing laminate thickness. The perforation energy of the 2 mm-thick laminate is only approximately 49.7 J. When the thickness increases to 4 mm, the perforation energy reaches approximately 120 J. As the thickness further increases to 8 mm and 10 mm, the energy required for perforation increases to approximately 321.4 J and 457.7 J, respectively.

## 6. Conclusions

This study systematically investigates the static mechanical performance, low-velocity impact response, and damage evolution characteristics of Triax-GF/PMMA thermoplastic composites through experimental tests and ABAQUS explicit dynamic simulations. The main conclusions are summarized as follows:

(1) Triax-GF/PMMA laminates were prepared via the VARI process using a PMMA/MMA binary resin system. A complete set of basic constitutive parameters, including modulus, strength, and shear properties, were acquired through quasi-static tensile, compressive and in-plane shear tests, providing fundamental mechanical data for numerical modeling of low-velocity impact.
(2) Low-velocity impact tests covering 15-120 J reveal that Triax-GF/PMMA possesses higher peak impact load and structural stiffness than conventional Triax-GF/Epoxy composites under identical fabrication and stacking conditions. Consistent enhancement is observed in post-impact compressive performance; specifically, the residual compressive strength of Triax-GF/PMMA is 9.8% higher than that of the epoxy counterpart at 50 J impact energy, demonstrating superior impact damage tolerance.
(3) A finite element model incorporating the Hashin progressive failure criterion was established to predict the impact behavior of triaxial fiber composites. The simulated peak loads deviate from experimental results by less than 8%, and the predicted damage morphologies match well with experimental observations. Simulation results show that high-stress regions of Triax-GF/PMMA are locally distributed along the 0°fiber direction under both non-penetration (30 J) and penetration (120 J) conditions. Compared with Triax-GF/Epoxy, the PMMA-based composite exhibits smaller deformation and damage severity, retaining better structural integrity under impact loading.
(4) Based on the validated numerical model, the perforation behavior of Triax-GF/PMMA laminates with thicknesses of 2 to 10 mm was further analyzed. The perforation energy increases nonlinearly with laminate thickness and follows a quadratic functional relationship. The fitted thickness-perforation energy prediction model shows good agreement with experimental data at 4 mm thickness, which can provide reliable references for the anti-impact structural design of Triax-GF/PMMA composites within the investigated thickness range.
(5) Previous studies have mainly focused on the processing characteristics, static mechanical properties, and recyclability of PMMA/MMA based glass fiber composites. The present study extends this understanding to the low velocity impact damage tolerance of triaxial laminates by integrating multidirectional mechanical characterization, impact testing, compression after impact (CAI) assessment, and experimentally validated progressive damage simulation. Future work should verify whether the observed performance advantages are retained under seawater or salt spray aging, combined moisture and temperature cycling, and repeated low velocity impacts, with particular emphasis on long term fiber/matrix and interlaminar durability. In addition, impact tests at more finely spaced energy levels across the transition region between 50 J and 120 J would help clarify the critical damage evolution and failure thresholds.

## Figures and Tables

**Figure 1:**
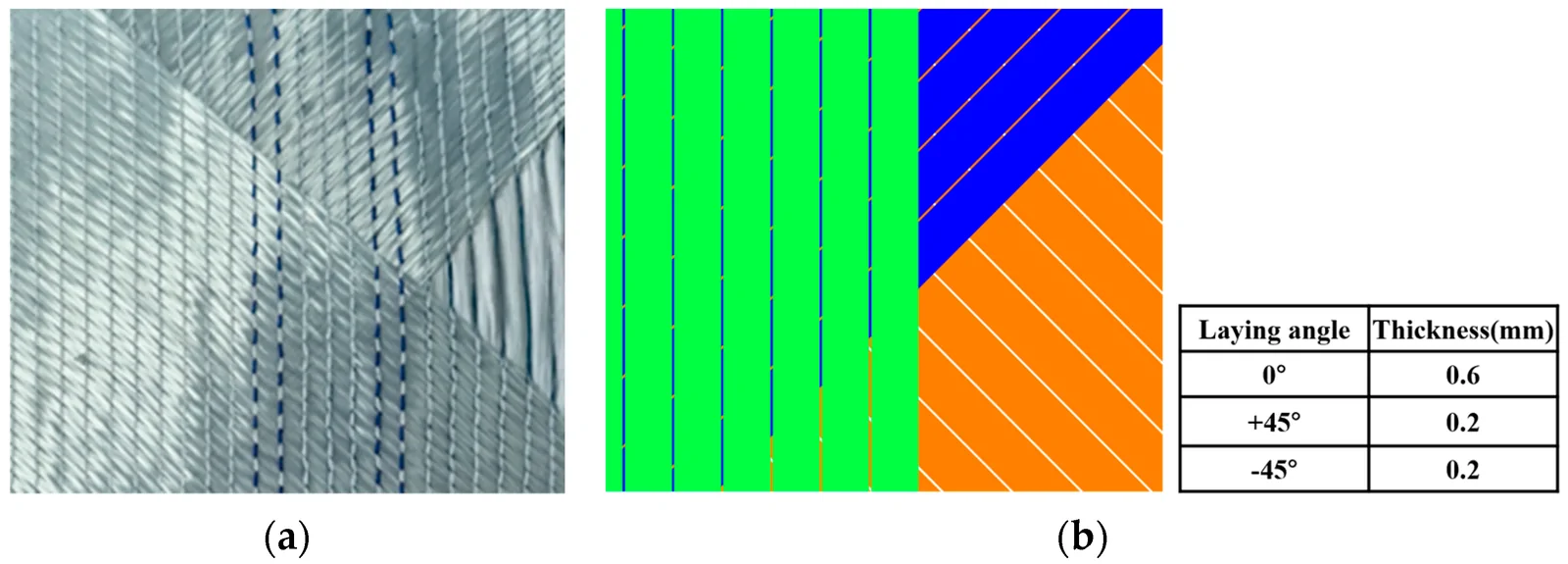
(**a**) Photograph of triaxial glass fabric; (**b**) schematic representation of its layered structure.

**Figure 2:**
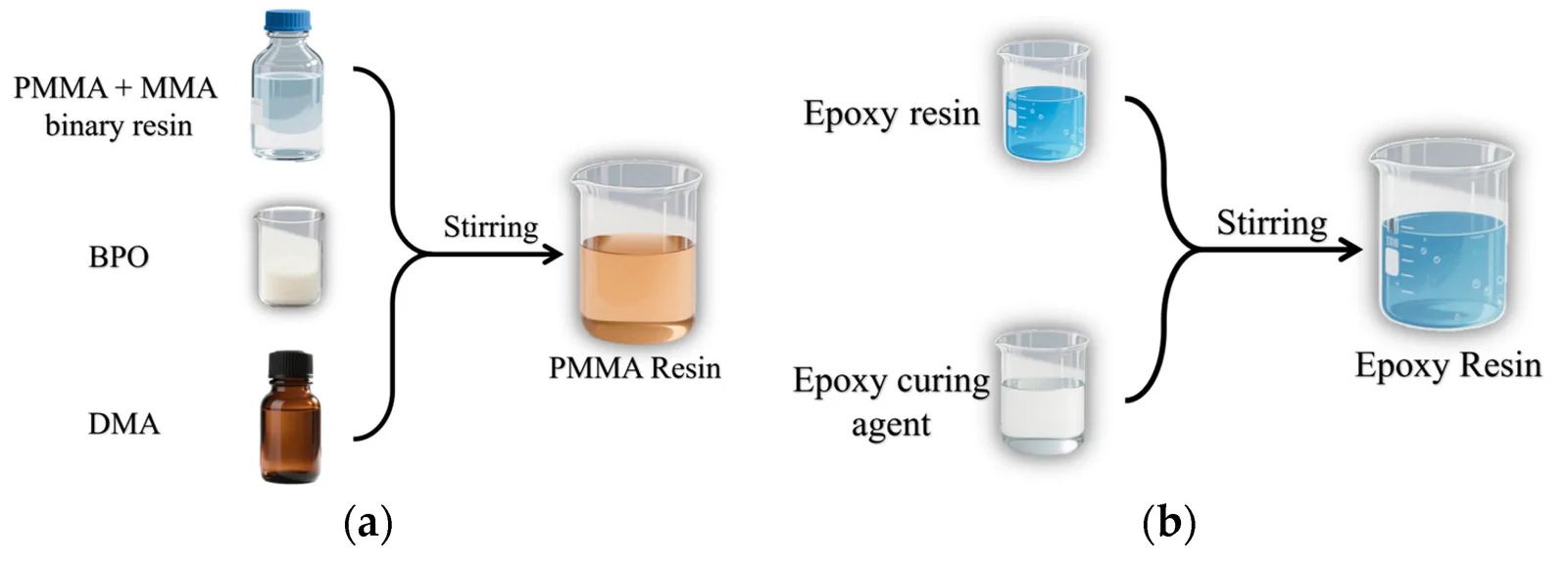
(**a**) Preparation of PMMA resin, Adapted from Ref. [27], Copyright 2026, with permission from Wiley. (**b**) Preparation of epoxy resin.

**Figure 3:**
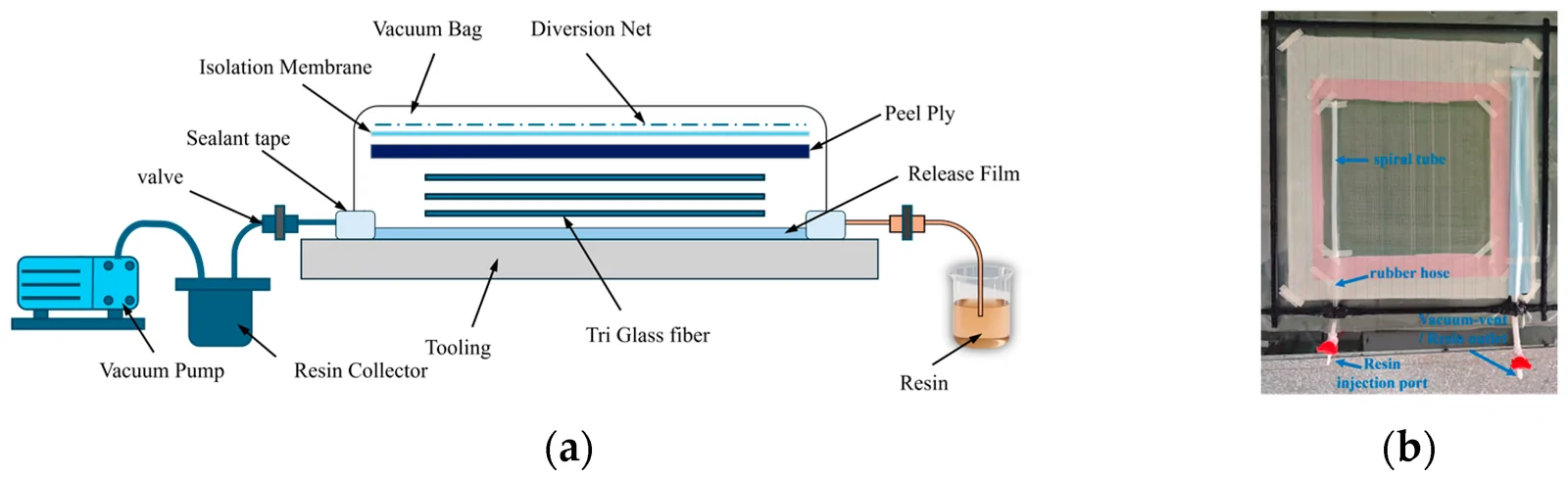
(**a**) Schematic of the VARI molding process for Triax-GF/PMMA; (**b**) photograph of the actual VARI mold. 3. Investigation on the Mechanical Properties of Triaxial Fabric Laminates.

**Figure 4:**
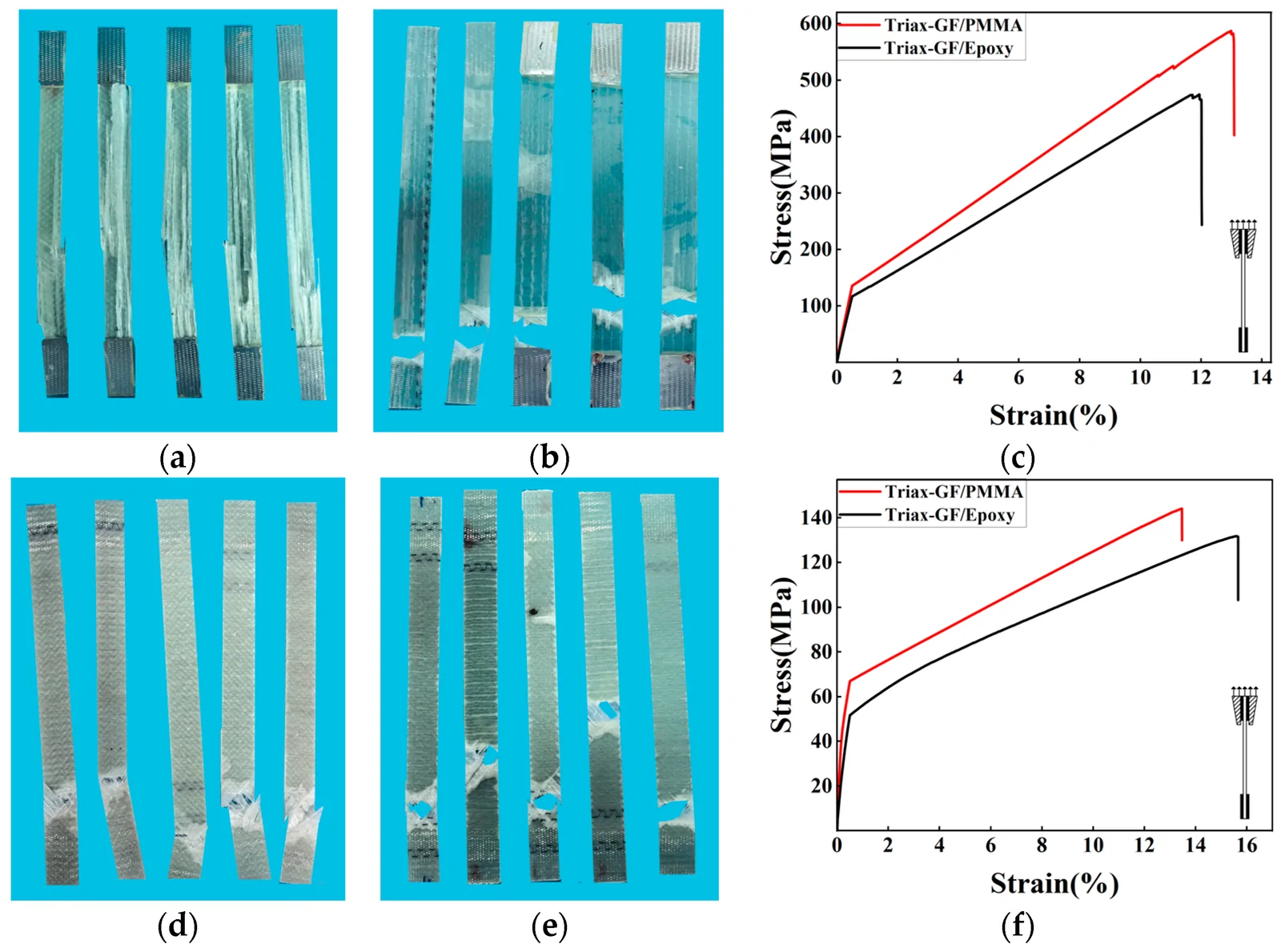
(**a**) Triax-GF/PMMA specimens after the 0° tensile test; (**b**) Triax-GF/Epoxy specimens after the 0° tensile test; (**c**) 0° tensile stress–strain curves; (**d**) Triax-GF/PMMA specimens after the 90° tensile test; (**e**) Triax-GF/Epoxy specimens after the 90° tensile test; (**f**) 90° tensile stress–strain curves.

**Figure 5:**
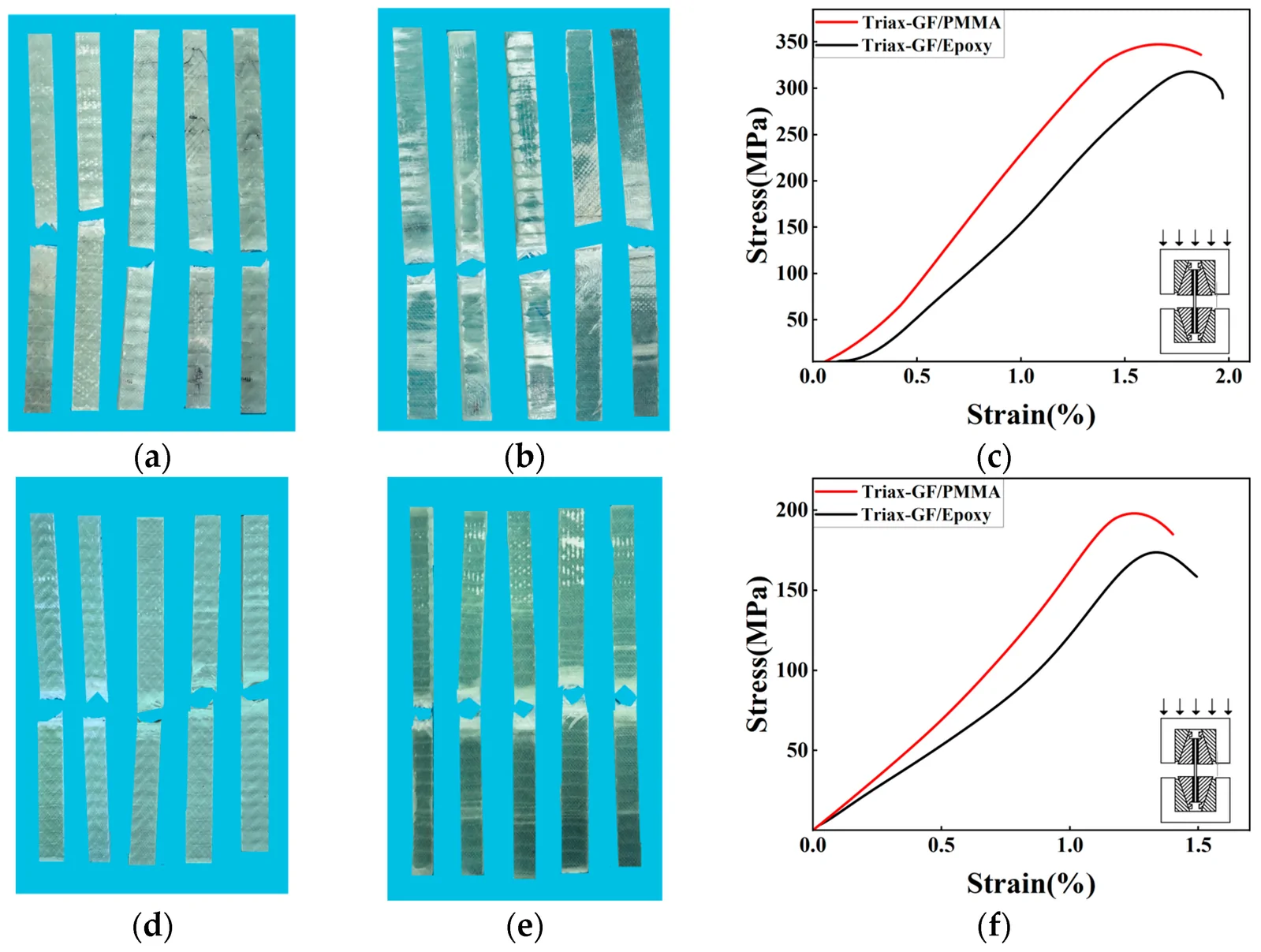
(**a**) Triax-GF/PMMA specimens after the 0° compression test; (**b**) Triax-GF/Epoxy specimens after the 0° compression test; (**c**) 0° compressive stress–strain curves; (**d**) Triax-GF/PMMA specimens after the 90° compression test; (**e**) Triax-GF/Epoxy specimens after the 90° compression test; (**f**) 90° compressive stress–strain curves.

**Figure 6:**
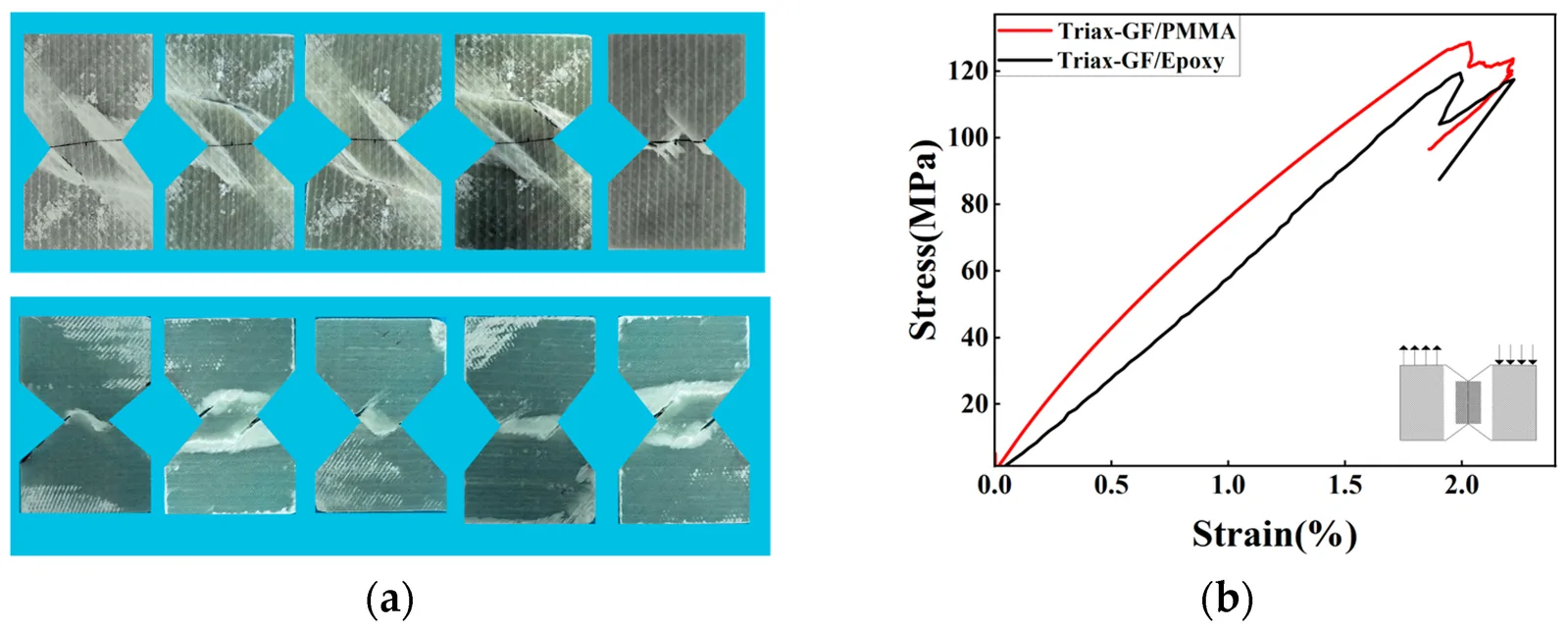
(**a**) photographs of Triax-GF/PMMA and Triax-GF/Epoxy specimens after testing; (**b**) in-plane shear stress–strain curves.

**Figure 7:**
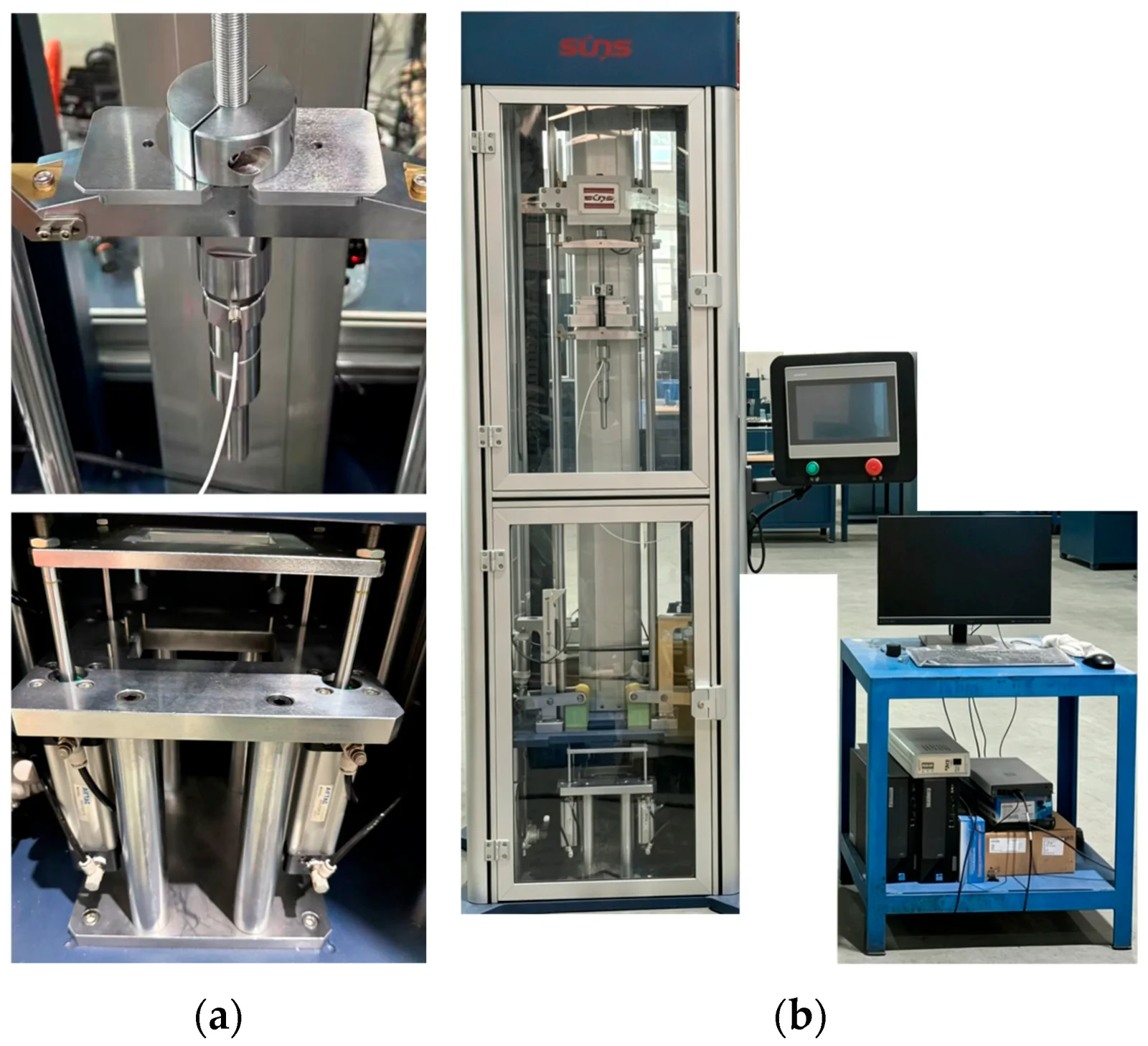
(**a**) Partial view of the impact fixture; (**b**) SANS low-velocity drop-weight impact test platform.

**Figure 8:**
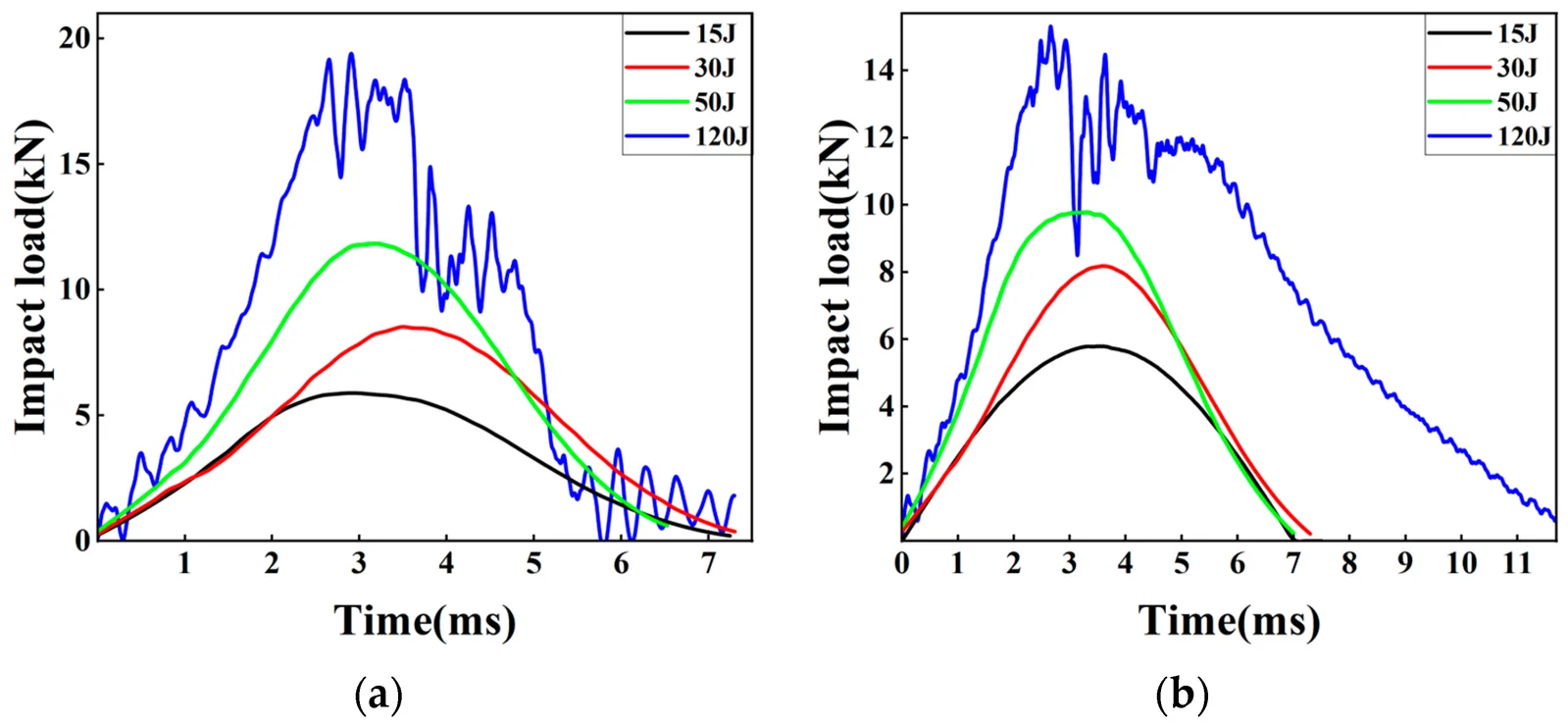
Comparison of force–time curves under different energy levels: (**a**) PMMA; (**b**) epoxy.

**Figure 9:**
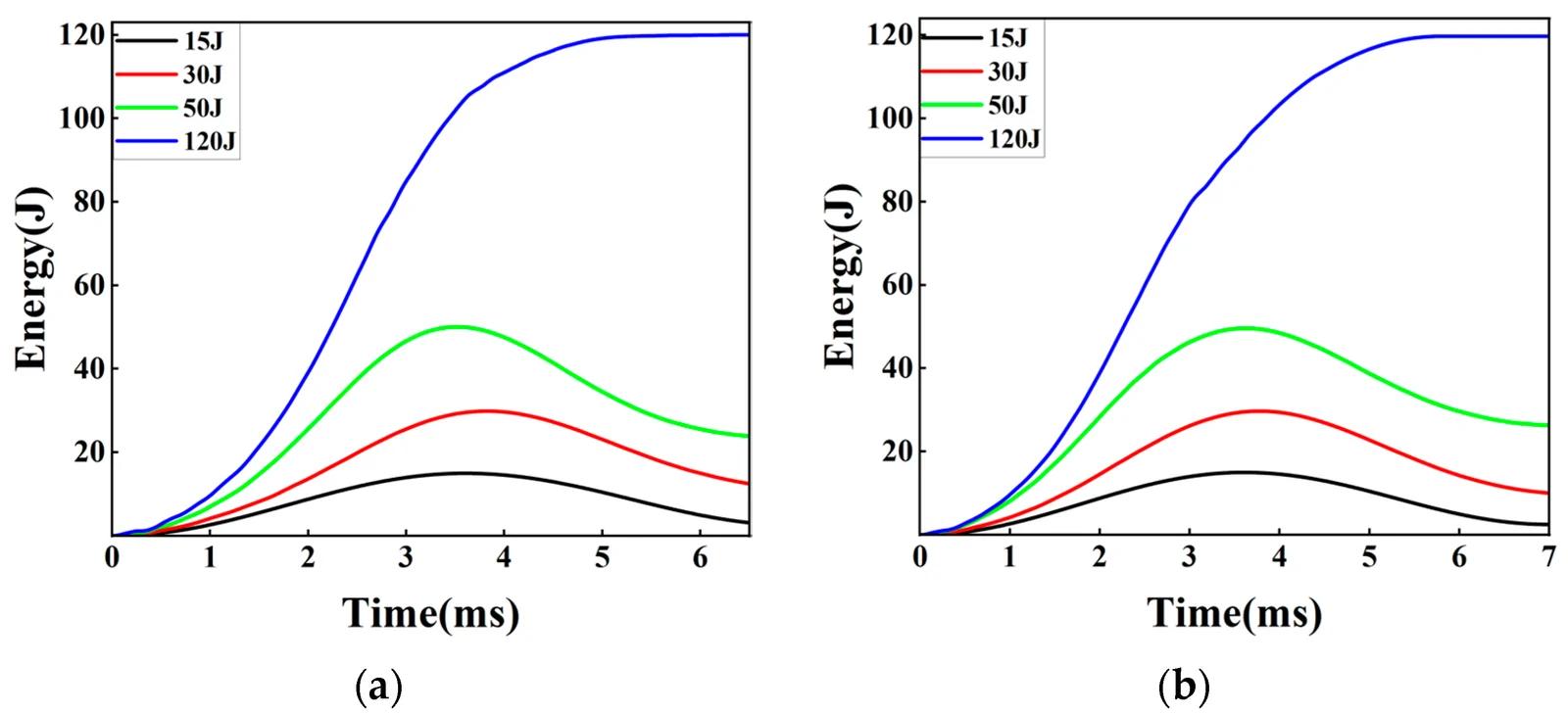
Comparison of energy–time curves under different energy levels: (**a**) PMMA; (**b**) epoxy.

**Figure 10:**
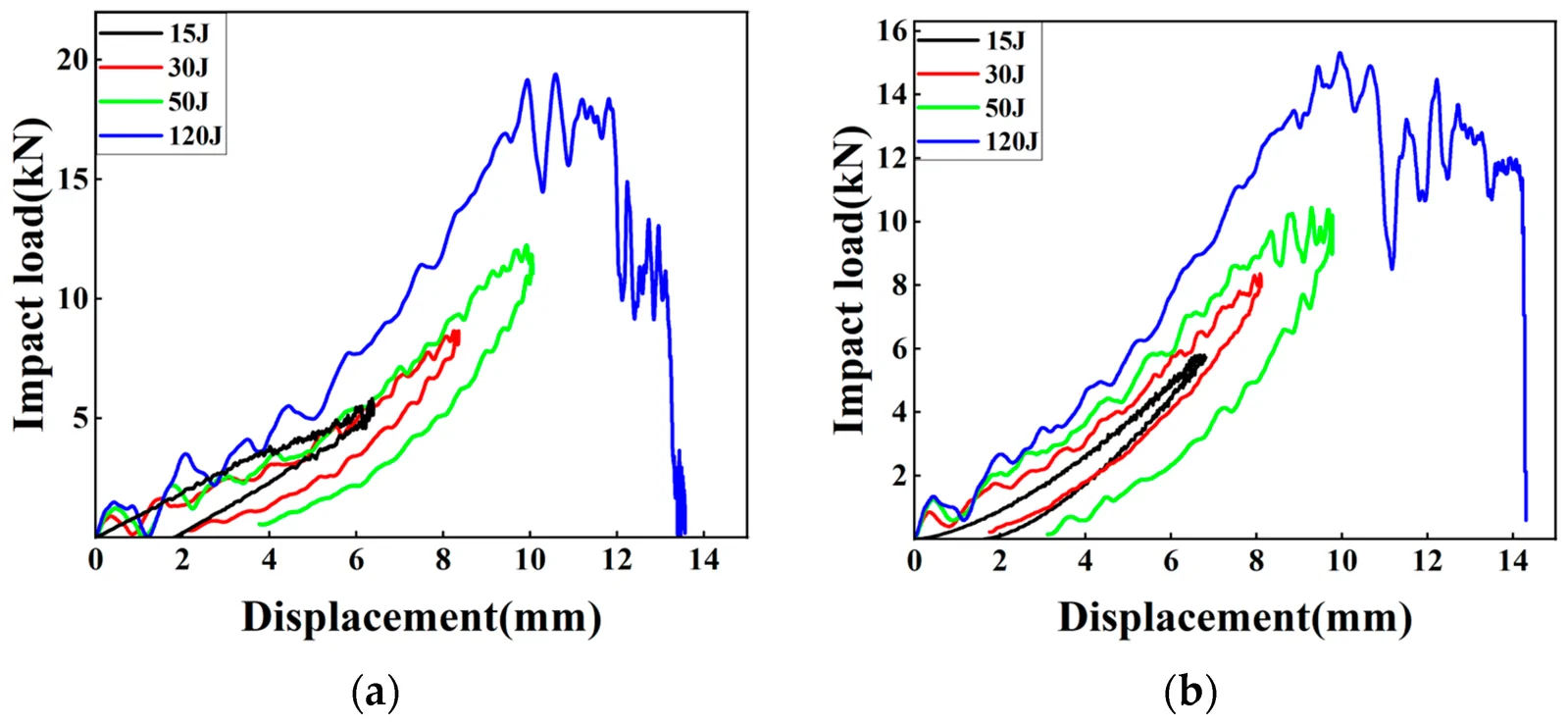
Comparison of force–displacement curves under different energy levels: (**a**) PMMA; (**b**) epoxy.

**Figure 11:**
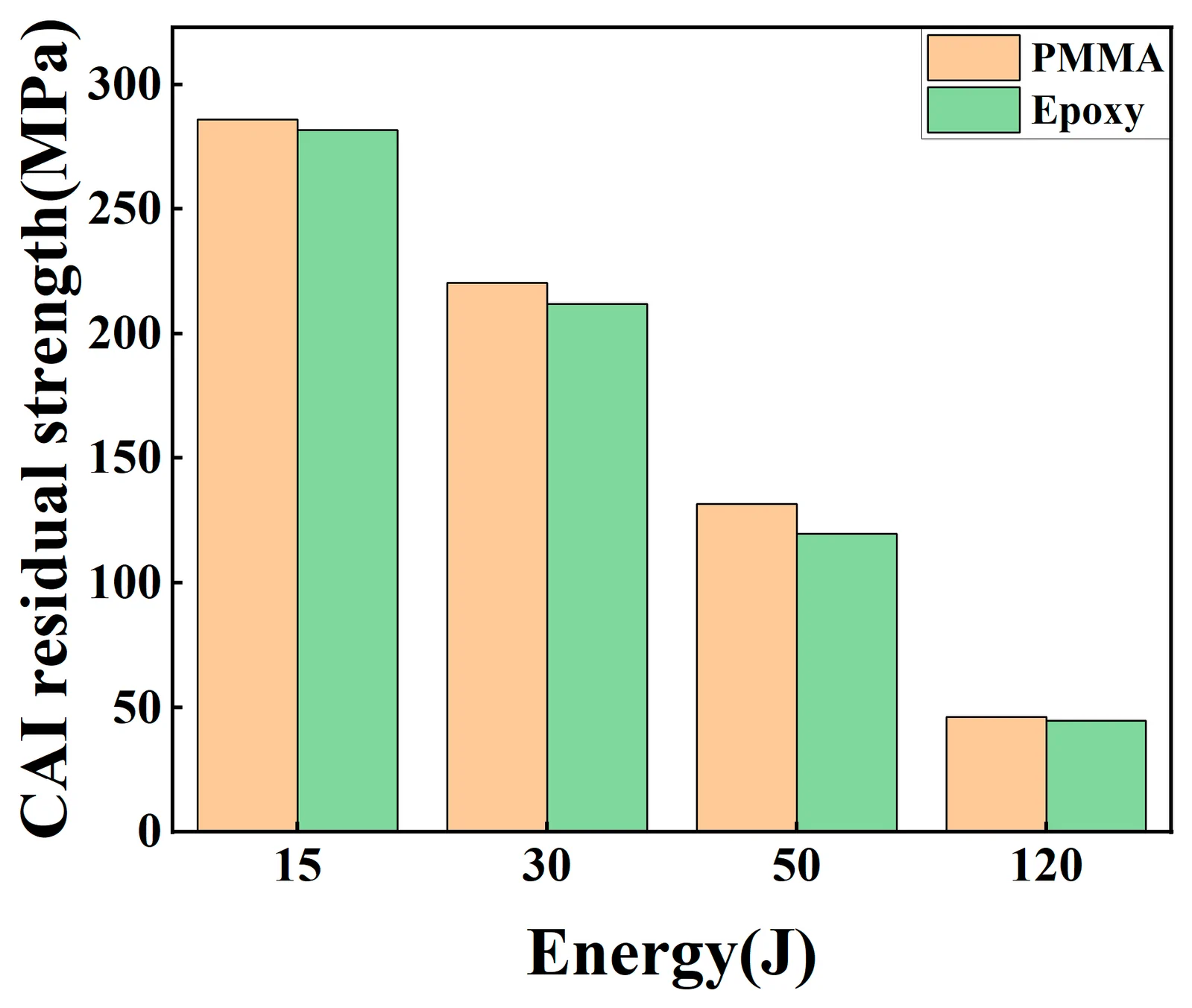
Ultimate residual compressive strength of the two materials under different energy levels.

**Figure 12:**
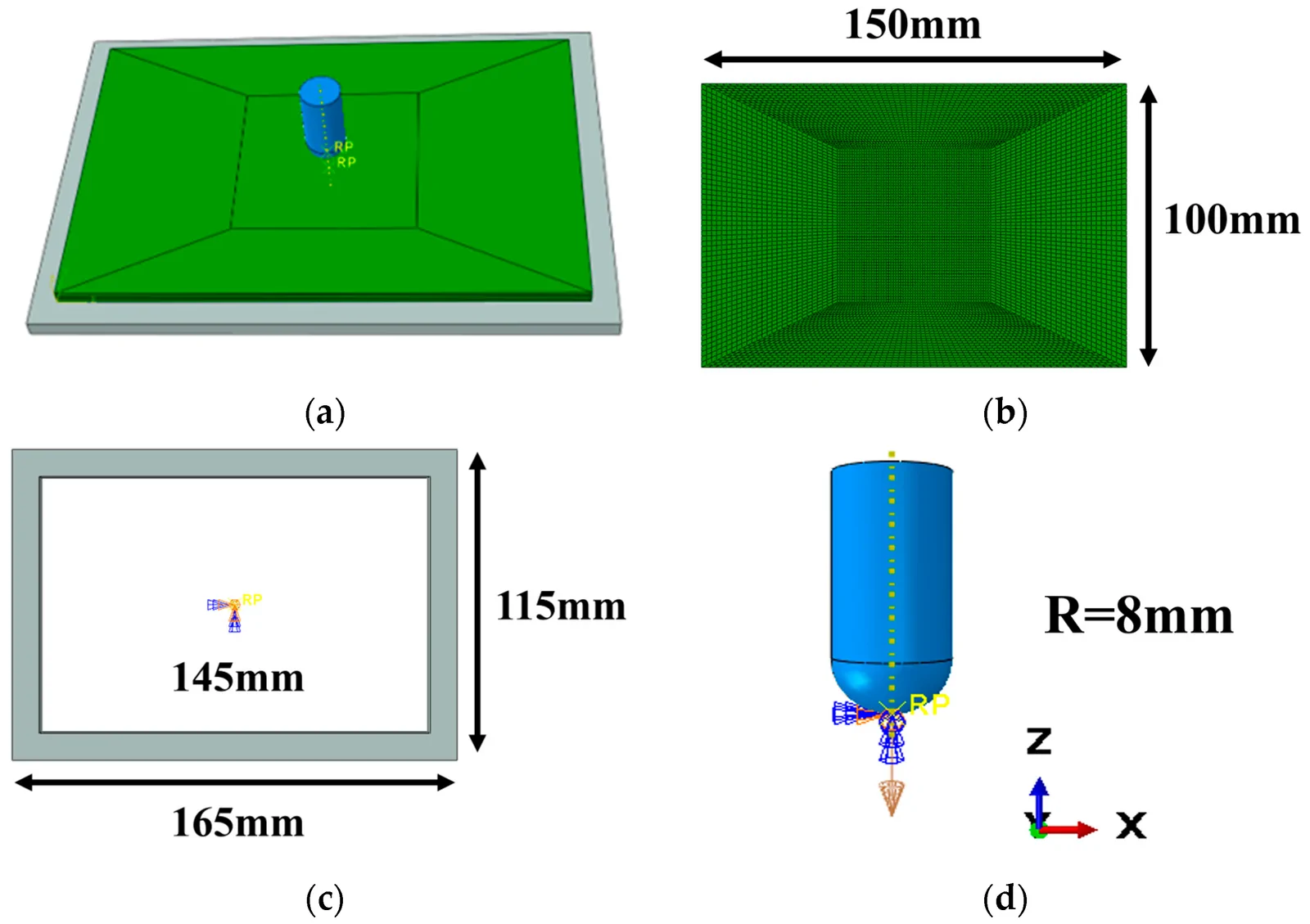
(**a**) Three-dimensional view of the simulation model; (**b**) mesh generation of the laminate; (**c**) boundary conditions; (**d**) constraints and loading of the impactor.

**Figure 13:**
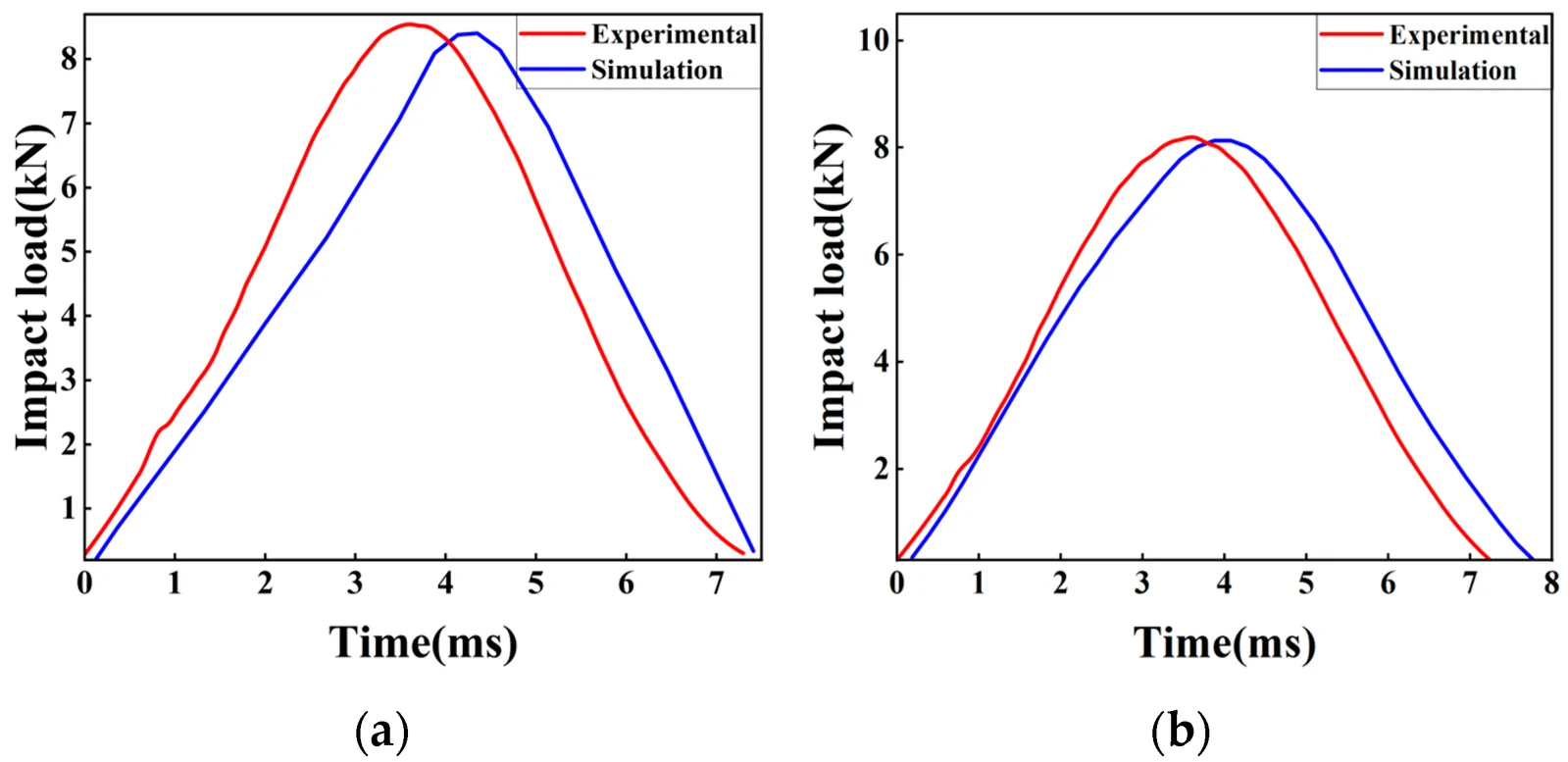
Comparison of simulated and experimental curves: (**a**) PMMA; (**b**) epoxy.

**Figure 14:**
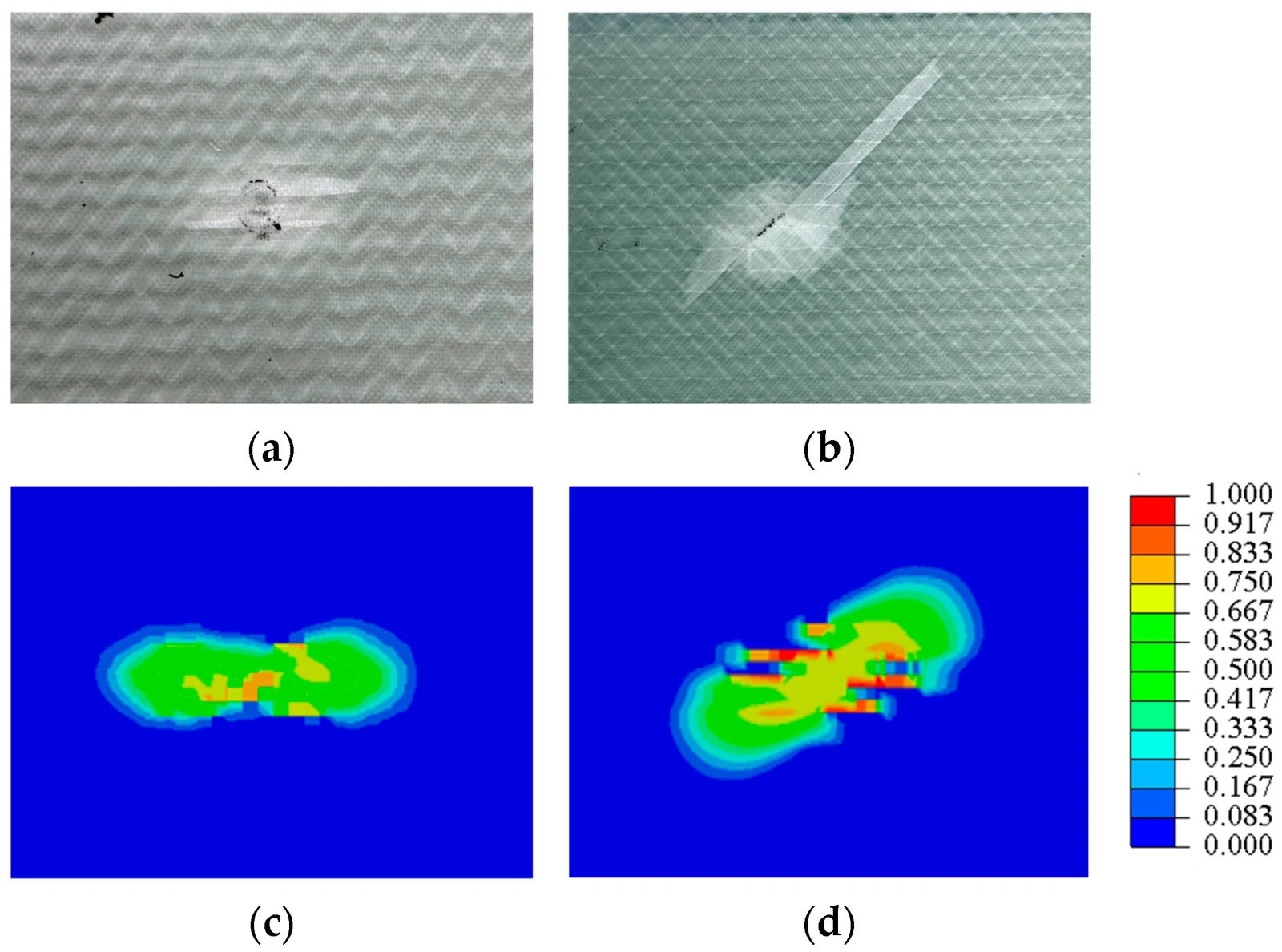
Validation of impact damage at 30 J. (**a**) Experimental impact face; (**b**) Experimental rear face; (**c**) Simulated EFC impact face; (**d**) Simulated EFT rear face.

**Figure 15:**
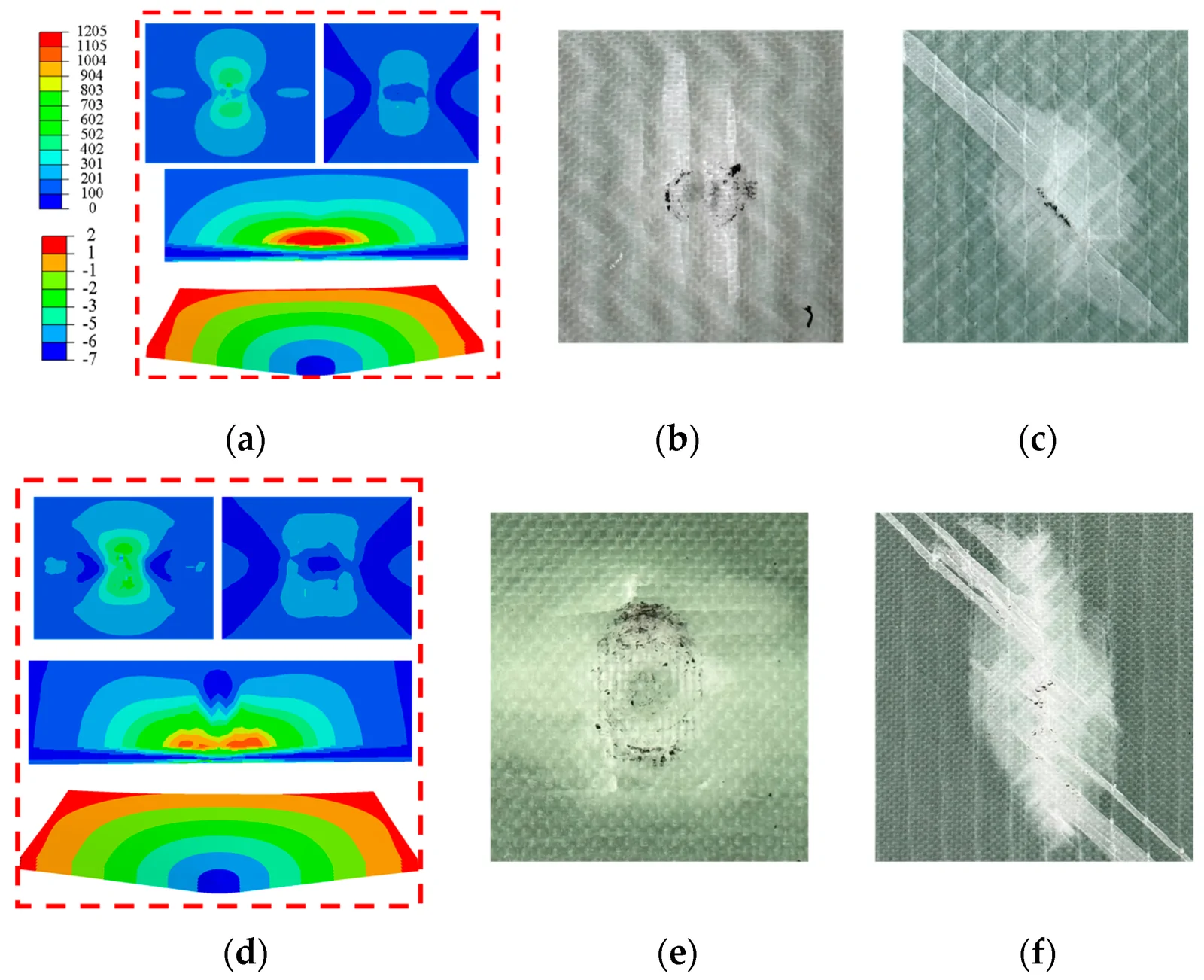
Impact damage at 30 J: (**a**) stress and displacement contours of Triax-GF/PMMA; (**b**,**c**) damage morphologies on the impact face and back face of Triax-GF/PMMA; (**d**) stress and displacement contours of Triax-GF/Epoxy; (**e**,**f**) damage morphologies on the impact face and back face of Triax-GF/Epoxy.

**Figure 16:**
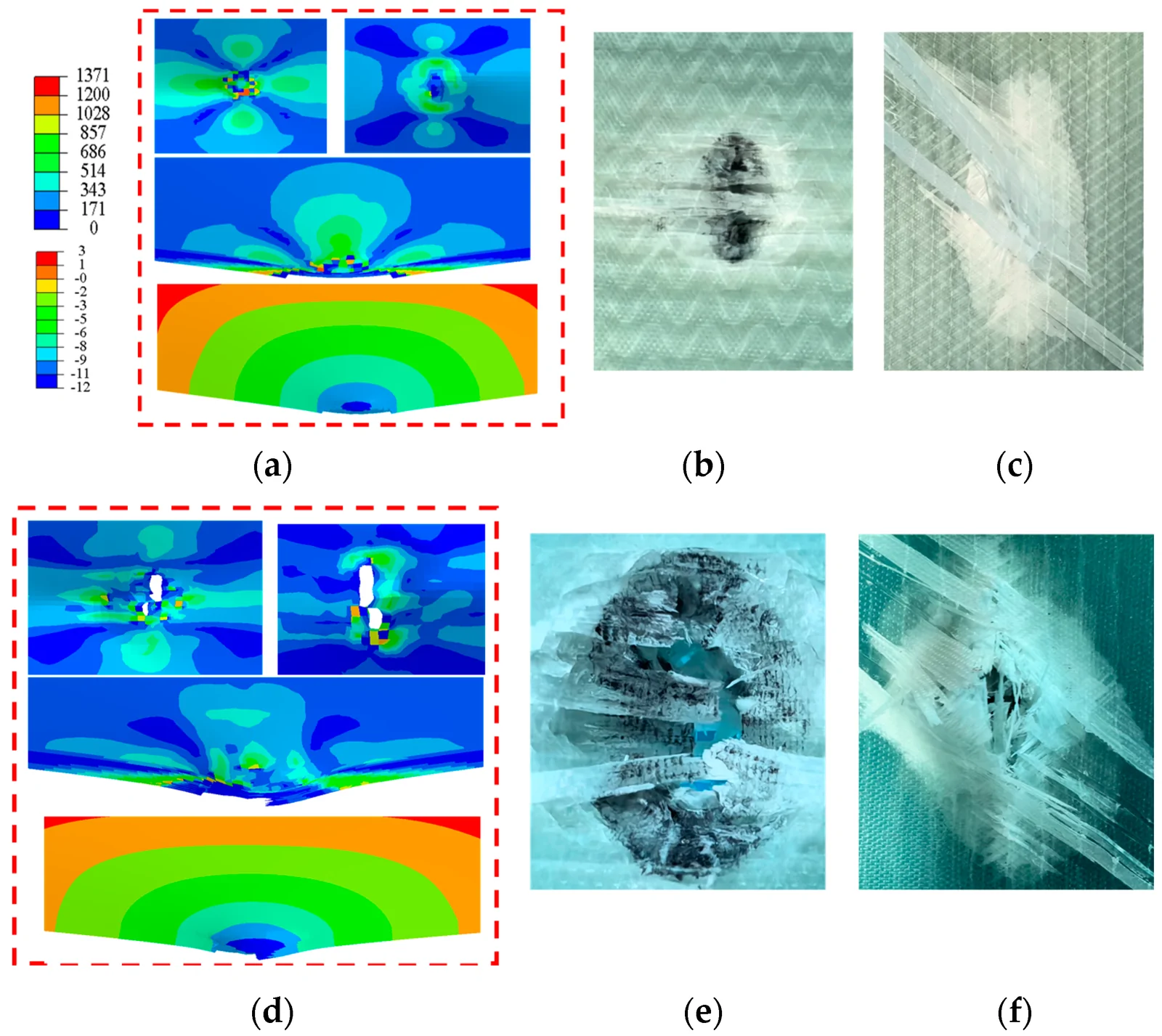
Impact damage at 120 J: (**a**) stress and displacement contours of Triax-GF/PMMA; (**b**,**c**) damage morphologies on the impact face and back face of Triax-GF/PMMA; (**d**) stress and displacement contours of Triax-GF/Epoxy; (**e**,**f**) damage morphologies on the impact face and back face of Triax-GF/Epoxy.

**Figure 17:**
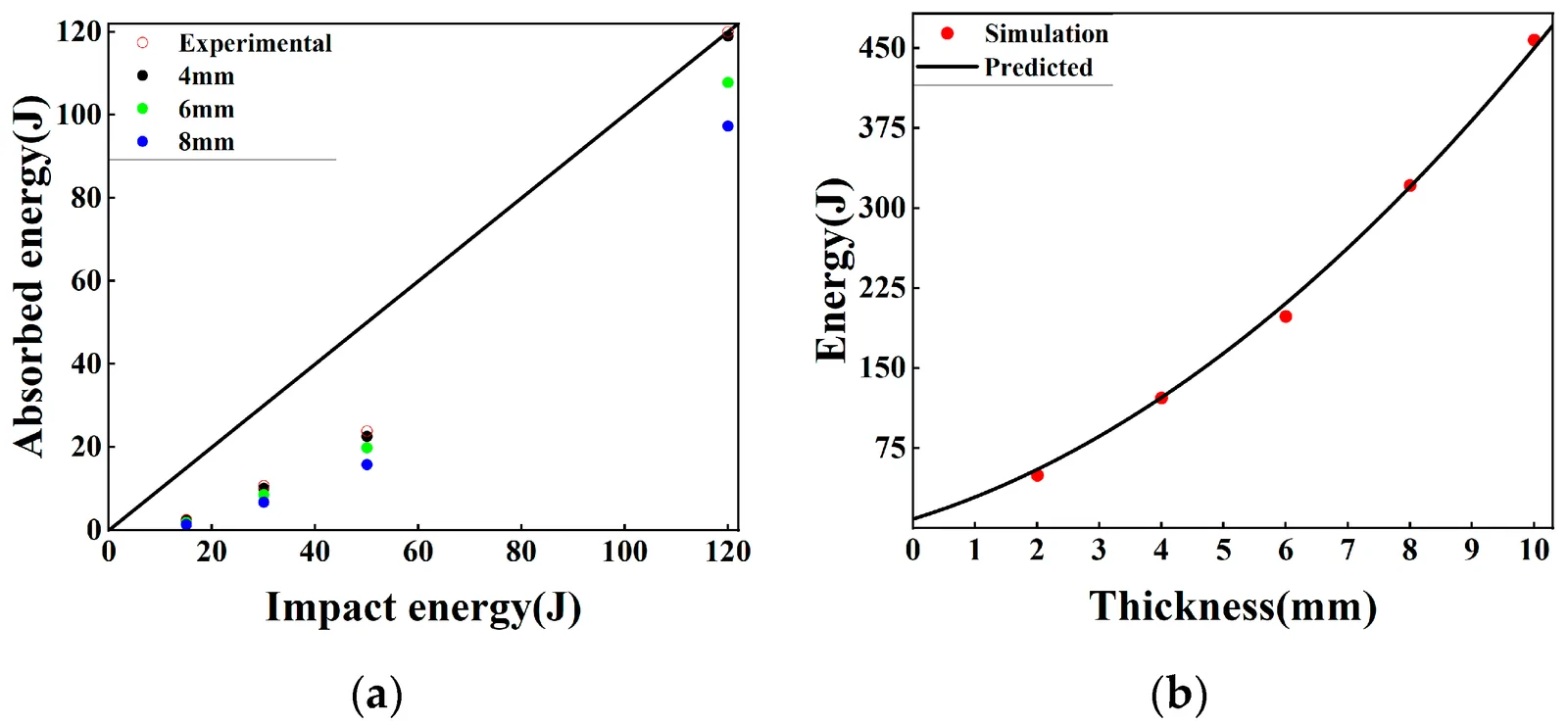
(**a**) Curves of input energy versus absorbed energy for different thicknesses; (**b**) perforation energy for different thicknesses.

**Table 1:** Parameters used for the calculation of fiber volume fraction.

Laminate	Specimen Dimensions (mm)	Average Thickness (mm)	Laminate Mass (g)	Glass Fiber Weight Fraction (%)	Density (g/cm^3^)	Fiber Volume Fraction (%)
Triax-GF/PMMA	150 × 100 × 4	4 ± 0.15	129 ± 0.2	73.83 ± 1.5	2.187	85.76
Triax-GF/Epoxy	150 × 100 × 4	4 ± 0.15	131 ± 0.2	72.44 ± 1.5	2.168	84.87

**Table 2:** Properties of Triax-GF/PMMA and Triax-GF/Epoxy.

Material Properties	Triax-GF/PMMA	Triax-GF/Epoxy
*E*_1*t*_ (GPa)	28.53	22.62
*E*_2t_ (GPa)	16.73	10.81
*E*_1c_ (GPa) *E_2c_* (GPa)	27.24 13.02	19.00 9.45
*V*_12_	0.28	0.30
*G*_12_ (GPa)	8.19	6.13
*G*_23_ (GPa)	3.4	3.1
*Xt* (MPa)	587.10	474.68
*Xc* (MPa)	347.49	317.79
*Yt* (MPa)	144.11	131.79
*Yc* (MPa)	197.48	173.57
*S*_12_ (MPa)	128.41	119.44
*ρ* (kg/m^3^)	2187	2168

**Table 3:** Cohesive-interface parameters used in the finite element simulations.

	Triax-GF/PMMA	Triax-GF/Epoxy
*t_n_*^0^ (MPa)	20.6	36.9
*ts*^0^ (MPa)	31.5	45.6
*G*_IC_ (N/mm)	0.48	0.86
*G*_IIC_ (N/mm)	1.09	1.43

**Table 4:** Comparison of experimental and simulated peak forces for Triax-GF/PMMA and Triax-GF/Epoxy under different energy levels.

Impact Energy (J)	PMMA		Epoxy	
	Exp(kN)	Sim(kN)	Exp(kN)	Sim(kN)
15	6.01	5.90	5.8	5.69
Deviation (%)	/	−1.83	/	−1.90
30	8.56	8.38	8.16	7.97
Deviation (%)	/	−2.10	/	−2.33
50	11.84	11.13	9.98	9.19
Deviation (%)	/	−6.00	/	−7.92
120	19.35	18.97	15.29	14.58
Deviation (%)	/	−1.96	/	−4.64

## Data Availability

The original contributions presented in this study are included in the article. Further inquiries can be directed to the corresponding authors.
